# An ensemble forecast system for tracking dynamics of dengue outbreaks and its validation in China

**DOI:** 10.1371/journal.pcbi.1010218

**Published:** 2022-06-27

**Authors:** Yuliang Chen, Tao Liu, Xiaolin Yu, Qinghui Zeng, Zixi Cai, Haisheng Wu, Qingying Zhang, Jianpeng Xiao, Wenjun Ma, Sen Pei, Pi Guo

**Affiliations:** 1 Department of Preventive Medicine, Shantou University Medical College, Shantou China; 2 Guangdong Provincial Institute of Public Health, Guangdong Provincial Center for Disease Control and Prevention, Guangzhou, China; 3 Shantou Center for Disease Control and Prevention, Shantou, China; 4 Department of Environmental Health Sciences, Mailman School of Public Health, Columbia University, New York, United States of America; University of Washington, UNITED STATES

## Abstract

As a common vector-borne disease, dengue fever remains challenging to predict due to large variations in epidemic size across seasons driven by a number of factors including population susceptibility, mosquito density, meteorological conditions, geographical factors, and human mobility. An ensemble forecast system for dengue fever is first proposed that addresses the difficulty of predicting outbreaks with drastically different scales. The ensemble forecast system based on a susceptible-infected-recovered (SIR) type of compartmental model coupled with a data assimilation method called the ensemble adjusted Kalman filter (EAKF) is constructed to generate real-time forecasts of dengue fever spread dynamics. The model was informed by meteorological and mosquito density information to depict the transmission of dengue virus among human and mosquito populations, and generate predictions. To account for the dramatic variations of outbreak size in different seasons, the effective population size parameter that is sequentially updated to adjust the predicted outbreak scale is introduced into the model. Before optimizing the transmission model, we update the effective population size using the most recent observations and historical records so that the predicted outbreak size is dynamically adjusted. In the retrospective forecast of dengue outbreaks in Guangzhou, China during the 2011–2017 seasons, the proposed forecast model generates accurate projections of peak timing, peak intensity, and total incidence, outperforming a generalized additive model approach. The ensemble forecast system can be operated in real-time and inform control planning to reduce the burden of dengue fever.

## Author summary

Dengue fever is one of the key neglected tropical diseases (NTDs) and threatens the health of billions of people worldwide. Nevertheless, accurate forecasts of dengue outbreaks remain difficult: the intensity of dengue fever outbreaks varies remarkably among different seasons and there are generally no cases or only sporadic cases of dengue fever outside the outbreak season. From the perspective of data distribution characteristics, the time series of dengue incidence are following zero-inflated distributions. Therefore, generating model forecasts using the type of zero-inflated data is usually beyond the predictive power of general discrete distributions such as Poisson distributions. These characteristics impose great challenges in accurate dengue predictions. We proposed to establish a combined forecast system based on susceptible-infected-recovered (SIR) model coupled with ensemble adjusted Kalman filter (EAKF) algorithm for depicting dengue transmission dynamics, thus accounting for the dramatic variations of outbreak size in different seasons. This approach allows for early warning of epidemics and outbreaks of dengue, and can inform control planning to reduce the burden of the disease.

## Introduction

**Fig 1:**
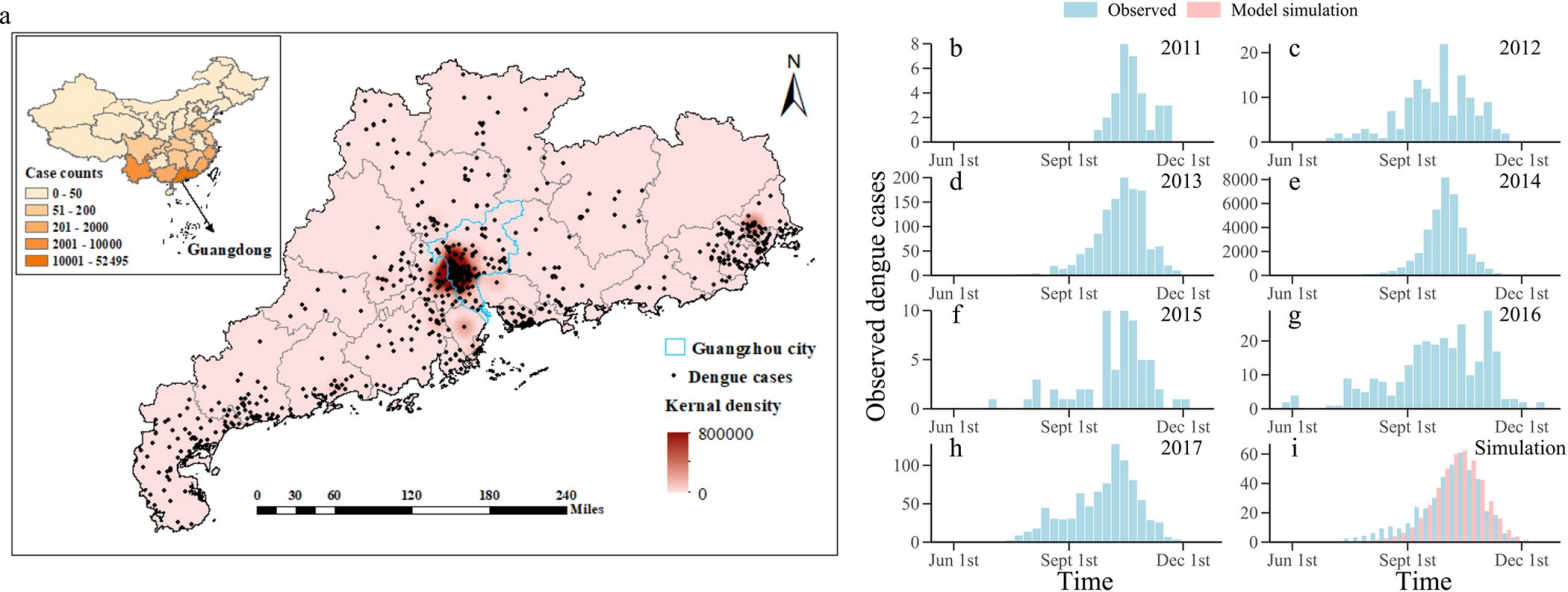
Seven consecutive dengue seasons (2011–2017) weekly observations and its geographical distribution, and free model simulation of dengue cases. **a** Geographical distribution of dengue cases and its kernel density estimation in Guangdong Province over the study period from January 2011 to December 2017. Cases counts and distinguished by colored according to the magnitude in each province also present (inset of **a**). In these 7 years, the cumulative number of dengue cases in Guangdong province accounted for 81% of the China mainland, of which Guangzhou took account for 76% of Guangdong province. **b-h** Weekly observations (from Jun 2011 to Dec 2017) of dengue cases in Guangzhou. **i** Average weekly observations (2011–2017, blue bar) and free model simulation (pink bar) of dengue cases. Only 35% of human cases had been reported around the season. *Base map sourced from the DIVA-GIS (https://www.diva-gis.org/gdata*).

Dengue fever, as one of the key neglected tropical diseases (NTDs) highlighted by the World Health Organization (WHO) [1], threatens the health of billions of people in the world. More than 390 million people are infected with dengue every year [2,3]. About 75% of the dengue burden is concentrated in Southeast Asia and the Western Pacific near the tropics. Guangdong province is one of the most prevalent areas in China. During the period of 2011–2017, dengue fever cases in Guangdong occupied 81% of the total cases in mainland China. Especially, Guangzhou city accounted for 76% of cases in Guangdong province (Fig 1A). In addition, with lengthened warm-season owing to global warming in recent years, mosquitoes have become more active, which increases the risk of Mosquito-borne disease outbreaks [4,5].

A super outbreak of dengue fever occurred in China during 2014, with the number of infected people reaching 46,864 [6]. If such a super outbreak could be predicted in advance, public health authorities can have more time to implement community prevention and mosquito control to contain the epidemic. However, accurate forecasts of dengue outbreaks remain difficult. The main characteristics of dengue fever prevalence and transmission in China are as follows: (1) the intensity of dengue fever outbreaks varies remarkably among different seasons. For example, the weekly number of newly infected dengue cases in Guangzhou city ranges from as low as 10 cases up to 8,000 cases at peak week; (2) there are generally no cases or only sporadic cases of dengue fever outside the outbreak season (as shown in Fig 1B–1H, the number of dengue fever cases in the non-outbreak period was basically zero around the year). The time series of dengue incidence usually present many zeros, and they are following zero-inflated distributions. Therefore, generating model forecasts using the type of zero-inflated data is usually beyond the predictive power of general discrete distributions such as Poisson distributions [7]. These characteristics impose great challenges in accurate dengue predictions.

Despite recent advances in real-time dengue prediction [8], few studies focus on forecasting systems developed especially for China. Existing forecast methods for infectious diseases generally fall in three categories: statistical approaches (e.g., time series analysis, Bayesian modeling averaging [9–12]), state-space estimation methods (e.g., model-inference systems [13–16]), and deep learning (neural network algorithm [17,18]). Recently, a number of model-inference systems have been developed and used to generate accurate ensemble forecasts for infectious disease. Coupling with data assimilation algorithms, the dynamic model can be calibrated to the observed data stream and generates a real-time prediction of infectious diseases, such as West Nile virus (WNV), Ebola, influenza, and other respiratory viruses [16,19–23].

In order to construct a model-inference system to generate real-time forecasts of dengue fever spread dynamics in China, the susceptible-infected-recovered (SIR) model, a dynamic model depicting dengue transmission, was adopted and coupled with reported human cases of dengue fever using the ensemble adjusted Kalman filter (EAKF) [23] for data assimilation. The combined SIR-EAKF system, informed by meteorological and mosquito density data, simulates the transmission of dengue virus between mosquitoes and humans. To account for the dramatic variations of outbreak size in different seasons, we defined an effective population size that was sequentially updated to adjust the predicted outbreak scale. This adjustment was embedded in each data assimilation step. We validated the forecasting system using retrospective forecasts of dengue outbreaks in Guangzhou during the 2011–2012 to 2017–2018 seasons.

## Results

### Spatial-temporal distribution of dengue cases

During 2011–2017 seasons, dengue cases in Guangdong province accounted for 81% of the total reported cases in China, with Guangzhou city being the most prevalent area. According to the spatial distribution of dengue cases in 2014 shown in Fig 1A, dengue cases were mainly distributed in the Pearl River Delta of Guangdong province, and other densely populated areas. The Pearl River Delta was the most prevalent area of dengue fever in that year, and in particular dengue fever posed a major public health threat in Guangzhou (areas outlined with blue border lines in the map). Besides, we publicly obtained the raw shape files from the DIVA-GIS (*https*:*//www*.*diva-gis*.*org/gdata**)*, and generated the maps in this study.

As shown in Fig 1B–1H, although the dengue fever epidemic in seven years has similar seasonality characteristics, the scale of dengue fever outbreaks varies significantly from year to year. We observe that 8000 cases occurred in the week with the peak incidence in 2014 while less than 10 cases in 2011, suggesting that the interannual epidemic scale of dengue is very different. In addition, according to the interannual epidemic curve of dengue fever, there are generally no cases or only sporadic cases of dengue fever in other time except for outbreak seasons. This means that the time series of dengue incidence is very sparse, usually following zero-inflated distributions.

**Fig 2:**
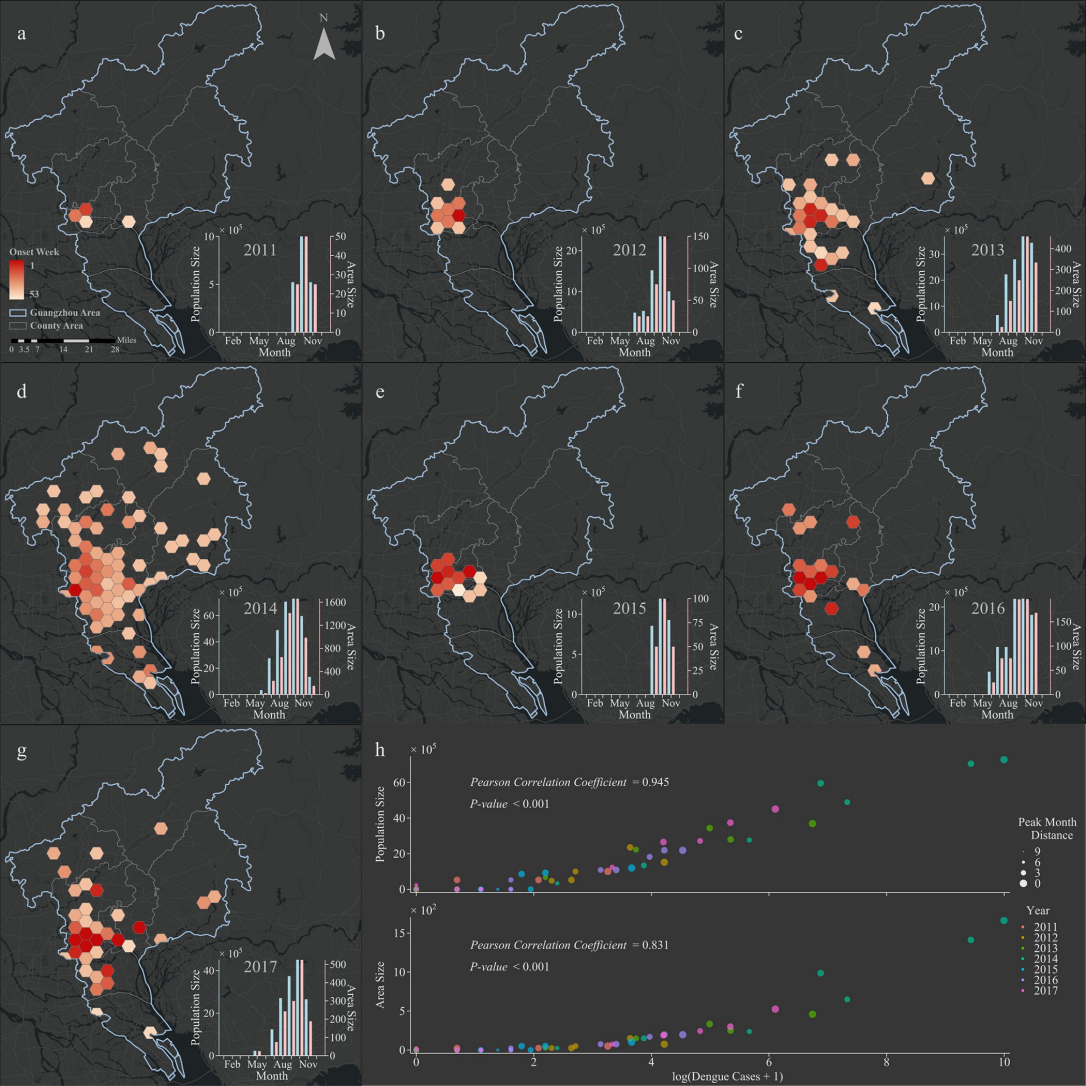
The spatial transmission pattern of dengue fever and its monthly changes of population size and area affected by the epidemic from 2011 to 2017. **a-g** The temporal and spatial spread of the epidemic in Guangzhou from 2011 to 2017. The onset week of administrative regions has shown. Small figures inset of a-g show the monthly change trend of population size and area affected by the epidemic. **h** Scatter plots displaying the relationship between monthly new infections and the population size and area affected by the epidemic. The distance of peak month and different years are respectively distinguished by the size and color of scatter. The results of Pearson correlation analysis are also shown. *Base map sourced from the DIVA-GIS (**https*:*//www*.*diva-gis*.*org/gdata**)*.

Besides, the distinct divergence of dengue seasons can be found from the spatial transmission in which the pattern of disease spread shows an obvious relationship between the epidemic-affected population size and the spatial distribution of infected cases. As shown in Fig 2A–2G, Guangzhou is subdivided into a number of hexagons with an area of 25 kilometers. The hexagons with color indicate the areas with at least 1 dengue case in the corresponding year. The onset week (time of first case) of dengue fever in the hexagon area is distinguished by color. The ongoing geographical expansion of dengue from the region where the first dengue infection occurs (onset area) to its periphery are similar for each year, which conforms to the pattern of adjacent space diffusion. The figure insets a-g are the distributions of the epidemic-affected population size (in blue color) and area (in red color) recorded in the epidemic regions (hexagon area). Assuming that the population is evenly distributed in space, the population size is calculated by the number of permanent residents in each county obtained from the China Statistical Yearbook (http://www.stats.gov.cn/tjsj/ndsj/). These figures indicate a clear pattern in both the population size and the area affected by the epidemic, with the time series of the number of new infected cases peaking in October.

Fig 2H represents a scatter plot of the logarithmic value of the number of newly infected dengue cases plus 1, the population size and area affected by the epidemic in each month during the period from 2011 to 2017. Different colors are used to distinguish the respective calendar year, and the distance to the peak time by month of the corresponding year is distinguished by the size of the points. As the observed peak time approaches, epidemic-affected population size and area increase each year. Furthermore, we performed a Pearson correlation analysis to measure the relationship between log(new infected dengue cases + 1) and the population size and area affected by the epidemic, respectively, and the results according to the Pearson correlation coefficients demonstrated that they were highly correlated. It suggests that the epidemic-affected population size as well as the scale of outbreak varies with the development of dengue in different years. As a result, in order to accurately predict and track dengue outbreaks with different scales, the combined model-EAKF system should incorporate a time-varying epidemic-affected population size (defined as the effective population size) instead of only using an identical fixed population size parameter.

### Construction of the combined SIR-EAKF and simulation of synthetic outbreak

Before introducing the effective population size into the forecasting framework for dengue fever, we constructed the coupled model-EAKF framework first. In order to better simulate the transmission dynamics of dengue fever, the SIR compartmental model for dengue incorporated the possibility of horizontal transmission of dengue virus between mosquitoes and human [24,25] and that of vertical transmission of dengue virus among mosquitoes [26]. Besides, we found a steady peak in dengue outbreaks, usually in October of a year. To understand the transmission dynamics, the annual average time series of mosquito birth rate (*μ*_*b*_(*t*)) and population transmission rate (*τ*(*t*)) were calculated respectively, and were incorporated in the compartmental model with dynamic states of infection. Specifically, a time-varying *μ*_*b*_(*t*) and a fixed death rate (*μ*_*d*_) can simulate the natural growth of mosquito population. And model parameter *τ*(*t*) can well explain the probability of successful transmission of dengue virus following a mosquito bite. Moreover, several studies [27,28] have proved that the reproductive capacity of dengue fever in mosquitoes is changing with the ambient temperature exposure, which is one of the reasons for the seasonal variation of dengue outbreaks.

To calculate *μ*_*b*_(*t*) in the transmission dynamics model, the mosquito oviposition index (MOI), a kind of surveillance data of mosquito density, was converted into mosquito natural birth rate by the following procedure. First, the MOI was smoothed by Fourier transform method, a method to analyze signals and reduce noises. Assuming that the mosquito population was the same at the beginning and the end of dengue season, the standardized natural growth proportion sequence (*MOI*^*a*^) can be obtained by *MOI*/MOI*, where MOI* was the inflection point of MOI in which the first derivative is the minimum. We assumed the oviposition period of mosquitoes was 16 days [29], so the natural birth rate was calculated by $\sqrt[{16}]{MOI^{a}}-1$. Finally, the *μ*_*b*_(*t*) can be calculated as the natural birth rate minus the constant mosquito death rate *μ*_*d*_. The relationship between ambient temperature and population transmission rate was represented by a piecewise linear function based on Liu et al. [27]. The function is listed as follows:

$$f(T)=\{\begin{array}{cc} 0 & T\leq 18\\ (T-18)\times 8/3 & 18<T\leq 23\\ 40/3+(T-23)\times 16/3 & 23<T\leq 28\\ 40+(T-28)\times 20/3 & 28<T\leq 32\\ 200/3 & T>32 \end{array}$$ (1)

where *T* is the daily ambient temperature, and *f*(*T*) is the population transmission rate.

Then, we used the model free simulation to find a set of parameters that could represent the average weekly reported dengue cases for seven dengue seasons, and thus built the compartment model to well capture the dynamics of dengue outbreaks. The free simulation was initiated with all mosquitoes and humans susceptible and model parameters *D* = 6, *β*_0_ = 0.05, *U* = 0.25, *μ*_*d*_ = 1/15, and *α* = 1/500000. Fig 1I shows the free simulation of the annual dengue case from 2011 to 2017 (except 2014) by the dengue SIR model. A good representation of mean weekly estimate of reported dengue cases was produced by the parameter combination chosen in this simulation. The output from model simulation was taken as the initial target to test the optimization efficiency of the EAKF. The synthetic observation was generated through adding noise to the state variables and model parameters in the free simulation, defined as the ‘truth’. Throughout our study, all real-time forecasts were made by a system (called the combined SIR-EAKF framework) based on the SIR-EAKF coupled with an estimation method for determining the effective population size. In a nutshell, after updating the effective population size to provide enough susceptible populations, the SIR-EAKF framework re-assimilates weekly reported dengue cases observed in this season. S1 Fig shows the forecast results of synthetic outbreaks and the inference of unobserved state variables and parameters generated by the combined SIR-EAKF model. For state variables including *I*_*M*_, *I*_*H*_, *NewI*_*M*_ and *NewI*_*H*_, the method can reliably capture the trend of the dengue epidemic trajectory during the whole prediction process. The estimation of state variables including *S*_*M*_, *S*_*H*_ and *N* are lower than the truth at the beginning until more observations are assimilated to the 20th week. In addition, the estimates of model parameters including *D* and *β* are adjusted to the truth and fluctuate around it.

### Retrospective forecasts

**Fig 3:**
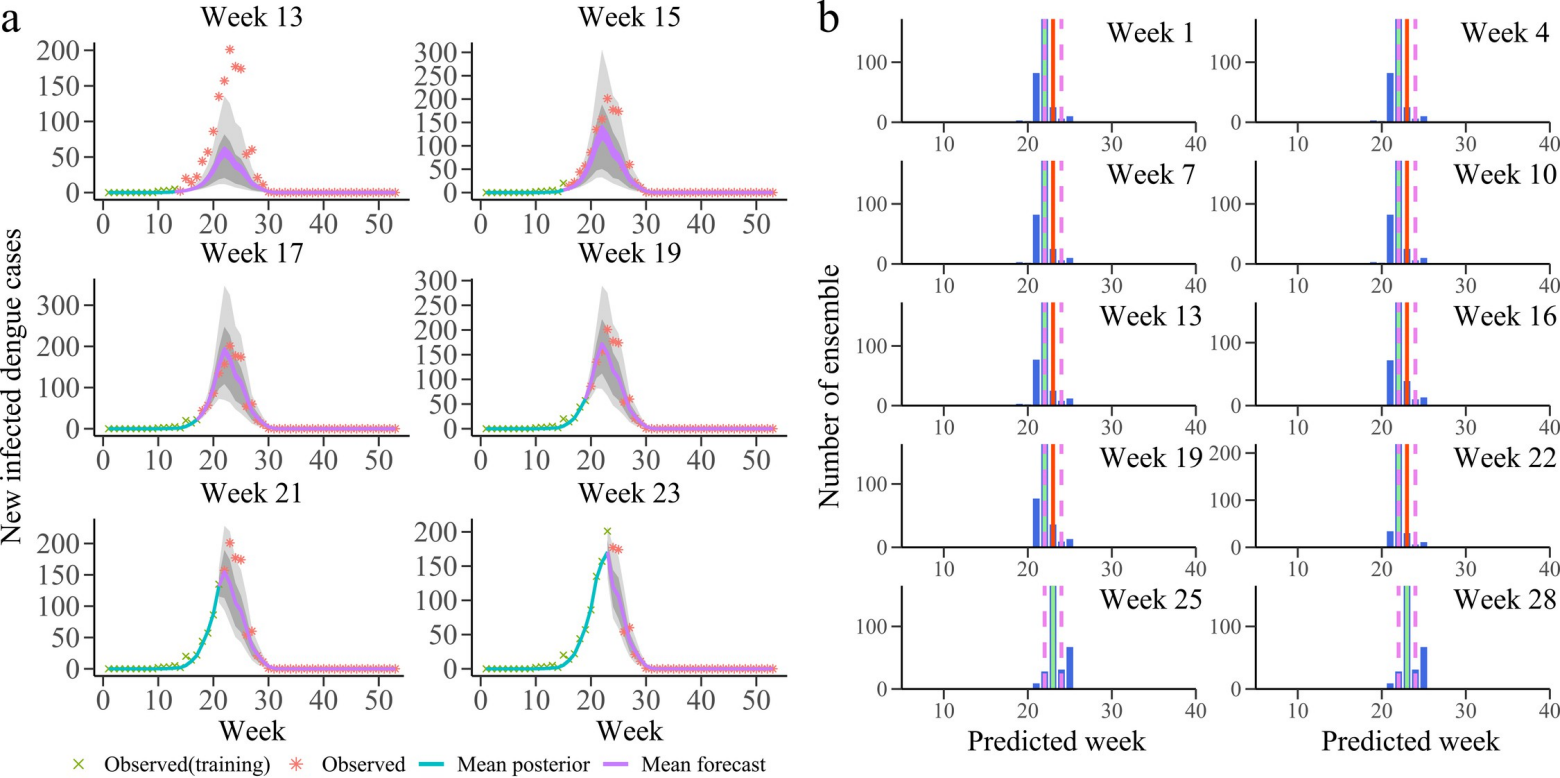
Results of 300-member combined SIR-EAKF retrospective forecasting for 2013–2014 seasons. **a** Ten bi-weekly forecast of dengue cases for 2013–2014 season. The purple lines are the ensemble mean forecasts, the grey area is the spread of the ensemble forecast (light grey represents area between 10th and 90th percentile and the darker grey area represents the spread between the 25th and 75th percentile), blue lines are the ensemble mean posterior distribution, green cross symbols are data points assimilated into the model and red star symbols are future observations. **b** Histogram of ensemble forecast peak time for prediction initiated at the end of weeks 1, 4, 7, 10, 13, 16, 19, 22, 25 and 28 week (blue). Also shown are the observed peak (red, week 23) and its ±1 week (pink) and the ensemble mean (green).

We used the combined SIR-EAKF system to generate retrospective weekly forecasts of dengue cases in Guangzhou from 2011–2017. In order to reduce the effect of filtering divergence, the forecast system started from the 20th week of each year and ends at the 19th week of next year. By integrating the time series of actual number of dengue cases (observation data stream) and ensemble of model simulations, the EAKF can better estimate ensemble state variables and model parameters until the end of the epidemic season. Fig 3A shows the successive forecasts of dengue cases during 2013–2014 seasons by the combined SIR-EAKF model. The trajectory of dengue outbreak predicted by the model better aligned with the observations before reaching the peak (the 23rd week of the epidemic season). With the assimilation of more observations, the ensemble variance within the model shrinks further. In addition, Fig 3B also shows the distribution of ensemble prediction of peak time. The predicted average peak time of dengue fever was within ± 1 week of the actual peak. More details of the successive forecasts of dengue cases during the 2013–2014 seasons are provided in S1 Video. The compartment-based vector-host model with seasonal characteristic signatures (e.g., natural birth and population transmission probability) exhibited a good performance, and the ensemble forecasts had successfully captured the whole dynamics of the dengue outbreak.

We evaluated retrospective forecasts for seven dengue seasons using three targets [15]: peak time, peak intensity and total incidence. Each target is deemed accurate if: the predicted peak time is within ± 1 week of the observed peak time; the predicted peak intensity is within ± 25% (± 1 case) of the observed peak intensity; the forecasted total incidence is within ± 25% (± 1 case) of the observed total incidence. Based on these conditions, the evaluations of three targets for seven dengue seasons are shown in S2–S4 Figs The combined SIR-EAKF framework has a good predictive performance in terms of the target of peak time across the entire period of prediction. In addition, the model developed in this study can accurately predict the intensity of the peak and the total incidence prior to the peak time of dengue fever outbreaks.

**Fig 4:**
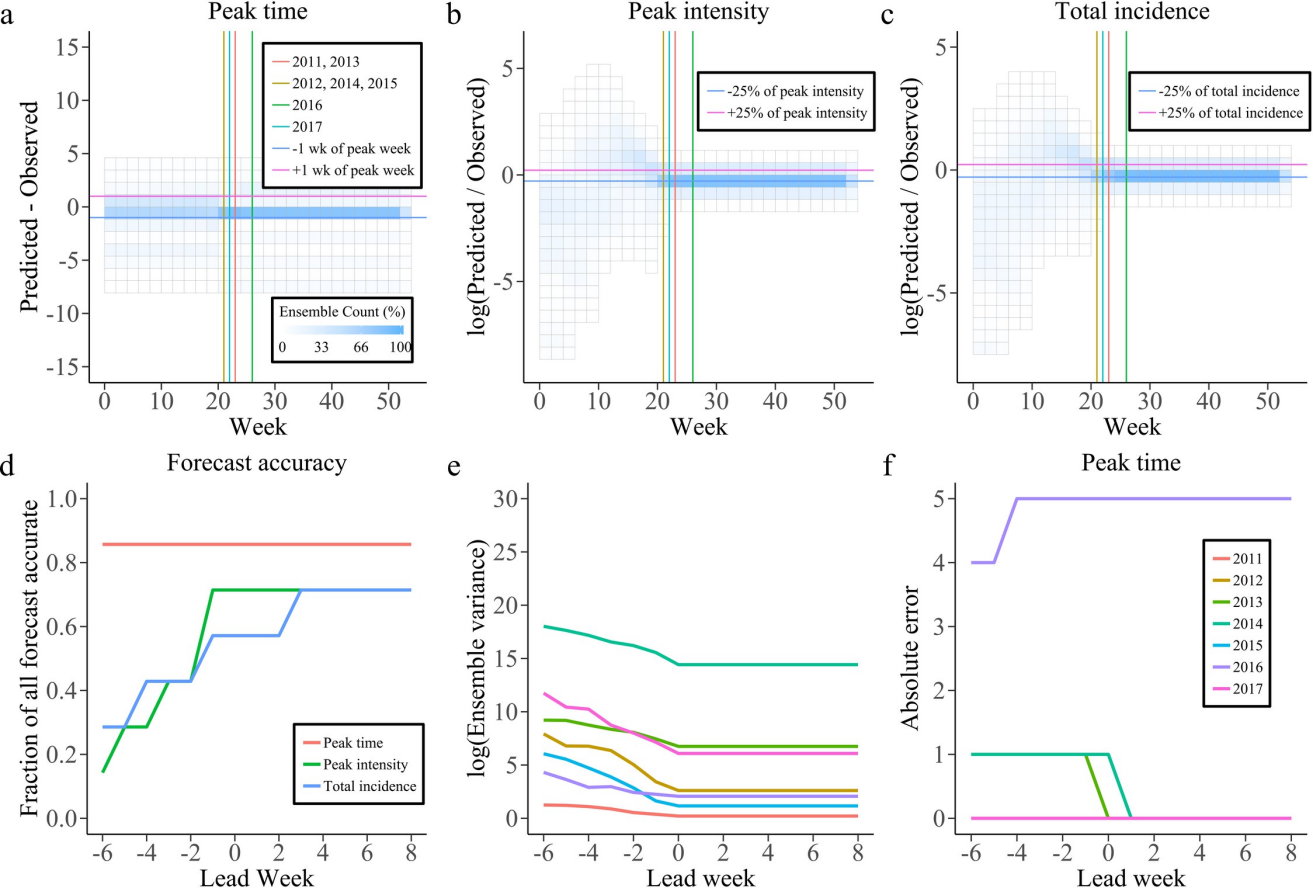
Results for 2011–2017 retrospective forecasts. **a-c** Distributions of the peak time (peak intensity and total incidence) distance from the predicted values to the observed target on each predicted week. The peak time for different dengue season is distinguished with distinct color vertical lines. The horizontal lines are representing the +1 wk (+25%, pink) and the -1wk (-25%, blue) from the observed peak week (peak intensity or total human dengue cases). **d** The fraction of forecasts accurate as a function of lead week for the targets peak time (red), peak intensity (green) and total incidence (blue). A forecast can be considered as accurate when (1) peak time was within ± 1 week of the observed peak of weekly new infected dengue cases; (2) peak intensity was within ± 25% or ± 1 cases of the observed peak weekly new infected dengue cases; (3) total incidence was within ± 25% of the observed. **e, f** characteristics the ensemble variance (**e**) and absolute error (**f**) for those predictions of each pandemic seasons which distinguished with different color.

Fig 4A–4C shows the ensemble distribution of the distance between prediction and observation of the three targets for seven dengue seasons. Most ensembles can accurately predict the peak time ahead of the observed peak time. For the peak intensity and the total incidence, with the accumulation of assimilation, ensemble distribution of the log(difference) gradually approaches zero when the predicted time is at or past the observed peak time. Finally, the fraction of forecast accurate for peak time, peak intensity and total incidence generated by the combined SIR-EAKF framework in seven dengue seasons is given. (Fig 4D). The forecast is grouped according to the predicted lead time, which is defined as the week of forecast generation minus the week of observed peak weekly dengue reported cases. Overall, forecasts of peak time were accurate > 85.7% of the time with 1 week prediction lead. Forecasts of peak intensity were also > 71.4% with one with ahead the observed peak time. For the total incidence, 57.1% of forecasts were accurate 1 week before the observed peak time, and > 71.4% of forecasts were accurate 2 week after the observed peak time.

**Fig 5:**
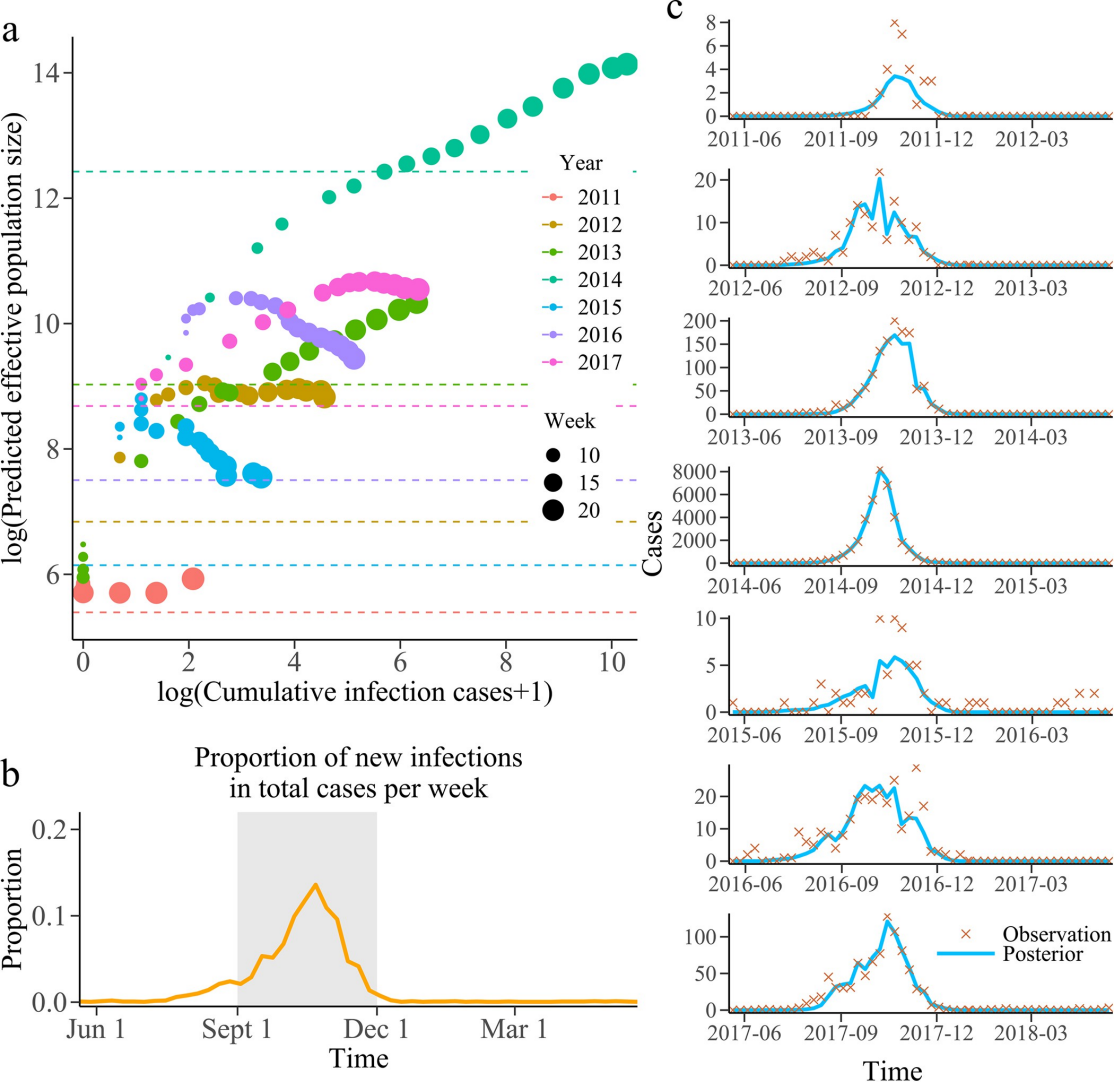
Prediction of population size and its function. **a** The population size set within the 5th and 22nd (the average peak time) weeks of each season. Each color represents corresponding year and the circle size indicate the predicted week. The dotted lines represents log(cumulative dengue cases over the corresponding season). The abscissa is log(cumulative infection number from the beginning of the dengue season to the current week +1). Besides, the ordinate is log(predicted population size). Based on the cumulative infection number from the beginning of the season to the current week, the total infection number was estimated as the population size of SIR-EAKF. For the convenience of display, log exchange was performed here. **b** Average proportion of new infections in total cases per week. The grey area shown that these 13 weeks (from 15 to 28 week) contain 90.7% of the total cases in the whole season except 2014–2015 season. **c** Weekly observed human dengue cases (cross symbols) for each year. The solid blue lines are the posterior mean of the combined SIR-EAKF fit.

Fig 5A illustrates the performance of estimating effective population size based on the estimation method of effective population size. The longitudinal value corresponding to the circle (the predictive value of the effective population size) should be higher than the total number of infections (indicated as dashed lines) at the end of the season. With the increase of data assimilation, the effective population size of the forecast system for each dengue season can be estimated to a sufficient value before the peak of dengue fever outbreak reaches. If and only if all susceptible people were infected during the season, the population size was equal to the total number of dengue cases reported. It can be seen in Fig 5A, after the 13th week of each dengue season, the value of effective population size parameter was estimated to be higher than the total number of dengue cases observed. The SIR-EAKF can accurately capture epidemic trends and estimate epidemic intensity when the parameter estimated by the proposed model was adequately larger than the minimum required number of total infections at the end of the season. Thus, the framework can successfully predict dengue epidemics on different scales. As shown in Fig 5C, the posterior ensemble mean estimated by the combined SIR-EAKF can predict dengue outbreaks well, except for the seasons with low epidemic size. Moreover, the analysis results of ensemble variance grouped by lead time (Fig 4E) indicate that the ensemble variance with more assimilated observations has a downward trend. Fig 4F shows the absolute difference between the ensemble average peak time and the observed peak time in seven dengue seasons grouped by lead time. The peak time in most of dengue seasons can be accurately predicted 6 weeks or more in advance, except for the peak time in the 2016–2017 dengue seasons because there were two peaks in the dengue epidemic.

**Fig 6:**
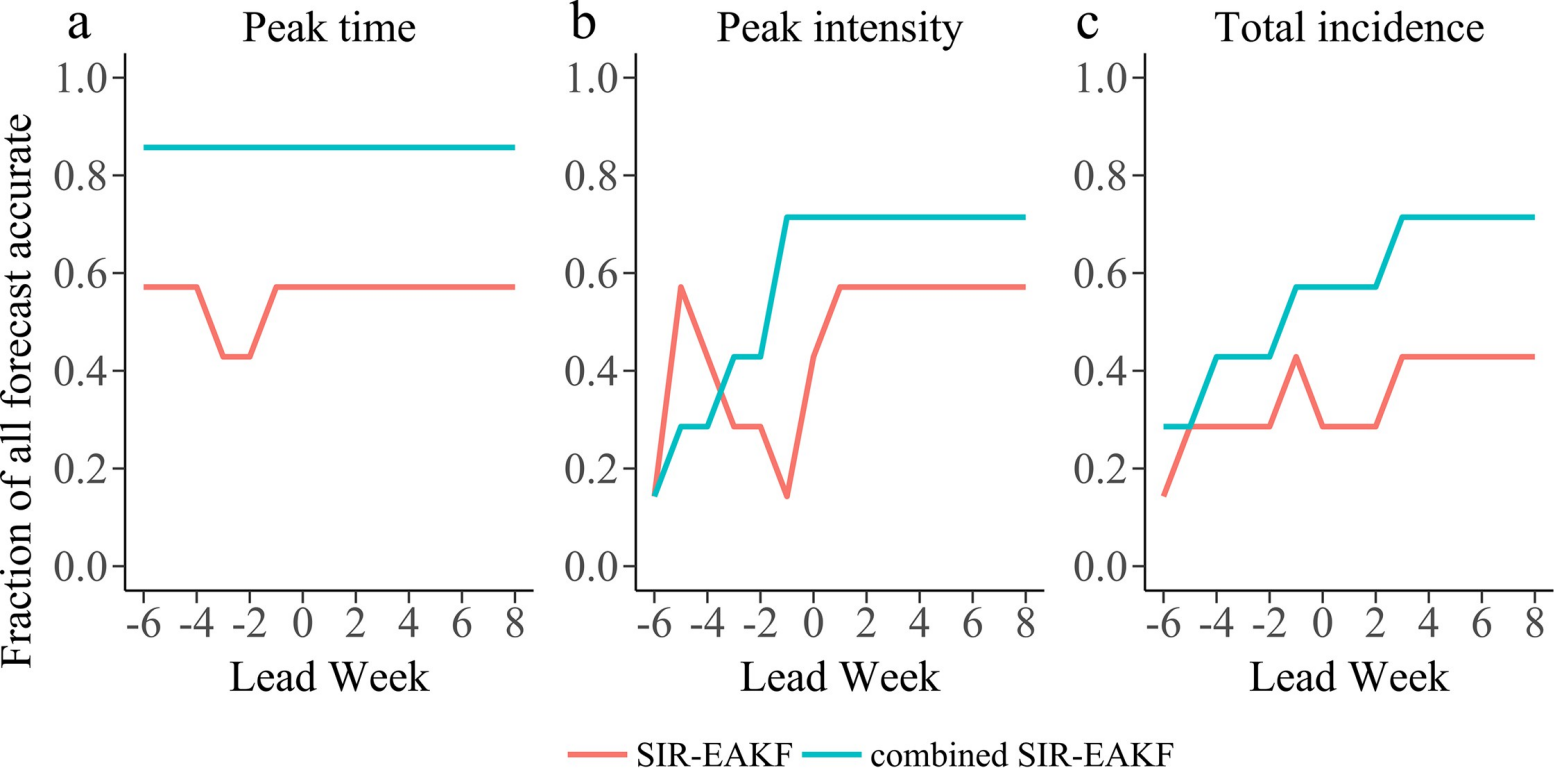
Comparison of the SIR-EAKF and the combined SIR-EAKF system. Historical dengue seasons of 2011–2012 to 2017–2018 were used to compare the prediction accuracy of two models, the SIR-EAKF model (red) and the combined SIR-EAKF model (blue). Finally, the fraction of forecasts accurate as a function of lead week for the targets peak time, peak intensity and total incidence.

To illustrate the advantage of the SIR-EAKF coupled with the dynamic adjustment of effective population size, we compared the performance of forecasting the 2011–2012 to 2017–2018 dengue seasons using the proposed model and the traditional SIR-EAKF model with a fix population size (for example, 300,000, which is sufficient for each season). As shown is Fig 6, the effective population size estimation helps improve the prediction accuracy of three targets by nearly 20%. In terms of peak time and total incidence, the performance of the combined SIR-EAKF framework is better than the traditional SIR-EAKF model across the entire period of model prediction. The prediction performance of the former is better than that of the traditional SIR-EAKF model for the target of peak intensity from the time point around the peak value.

To demonstrate robustness of the performance superiority, we extend our analysis to other 9 dengue fever prone cities in Guangdong, including Chaozhou, Dongguan, Foshan, Jiangmen, Qingyuan, Shantou, Shenzhen, Zhuhai and Zhanjiang. Due to the different geographical location, climate conditions and other factors, the patterns of dengue epidemic are various. Therefore, we used a wide range of parameter settings in the supplementary analysis. Each ensemble member state variables was initialized with $S_{M}(0)=N_{M}(0)-I_{M}(0)$, $I_{M}(0)=U(0,N_{M}/1000)$, $S_{H}(0)=N_{H}\times U(0.7,0.9)-I_{H}(0)$, $I_{H}(0)=U(0,1)$; and model parameters were randomly selected from uniform distributions: D=U(3,17), $\beta_{0}=U(0.025,0.075)$, $N=N_{H}=N_{M}(0)=N_{p}/U(0.6,0.8)$. The simulation was seeded with infected mosquitoes, *α*, during integration over season at a rate of 1 in 500,000. In addition, each ensemble member was initialized with constant model parameter: *U* = 0.25 and *μ*_*d*_ = 1/15 over an outbreak. Then, retrospective predictions were made based on the observed dengue cases from the seasons of 2014–2015 to those of 2017–2018. The prior and posterior mean ensemble predicted cases was shown in S8 Fig. We observed that the proposed model performed well for the forecasts of dengue epidemics of these 9 cities. The framework can almost capture the trajectory of the dengue outbreak except the very low-level dengue epidemic season (whose peak incidence was lower than 5 cases) in each season.

### Comparison between benchmark models and the combined SIR-EAKF framework

Previous studies have used the generalized additive model (GAM) or seasonal autoregressive integrated moving average (SARIMA) method to make a prediction of the outbreak of dengue fever and show a good performance [30–32]. We compared the prediction accuracy of GAM, SARIMA and SIR-EAKF using three targets (peak time, peak incidence, and total incidence) for the 2013–2014 through 2016–2017 dengue seasons. Following the study of Xu et al. [31], the GAM model was established in two steps. First, the generic model for vector and dengue, respectively, are given as follows:

$$V_{t}=a_{0}+f_{1}(V_{t-1})+f_{2}(P_{t-1})+\varepsilon_{t}$$ (2)

$$D_{t}=b_{0}+g_{1}(D_{t-1})+g_{2}(P_{t-1})+g_{2}(T_{t-1})+g_{4}(V_{t})+\delta_{t}$$ (3)

where *V*_*t*_ and *D*_*t*_ represent the weekly mosquito density and dengue incidence in week *t* respectively, *T*_*t*−1_ and *P*_*t*−1_ represent the weekly average of highest temperature and the number of days with rainfall for week *t*−1 respectively, the functions *f*_1_,*f*_2_ are linear functions, the functions *g*_1_,*g*_2_,*g*_3_,*g*_4_ are smooth functions of natural cubic spline with 3 degrees of freedom. Using the prediction of *V*_*t*_ from GAM of mosquito density, the GAM of dengue incidence then predicted the dengue incidence *D*_*t*_ in *t* week.

The above GAM can only forecast one week ahead and unable to forecast the rest of the dengue seasons. Consequently, the GAM was modified according to the number of lead weeks (*n*) as follows:

$$V_{t}=a_{0}+f_{1}(V_{t-n})+f_{2}(P_{t-n})+\varepsilon_{t}$$ (4)

$$D_{t}=b_{0}+g_{1}(D_{t-n})+g_{2}(P_{t-n})+g_{2}(T_{t-n})+g_{4}(V_{t})+\delta_{t}$$ (5)

Through the recursive prediction of GAM, the dengue cases can be predicted for remaining time of the season (*t*,*t*+1,…,53) at the *t*th week, which was used to compare with the forecast of combined SIR-EAKF model. To compare with the SIR-EAKF model, the similar posterior forecast was given by the fitting value of GAM model at week *t*. In this way, the GAM can predict the whole epidemic season at each week. Then, the predictive performance of GAM and SIR-EAKF was compared in terms of peak time, peak incidence and total incidence. The surveillance data range from the first week of observation to the current week was used to fit GAM recursively, by doing so, the forecasts for the remaining periods of dengue seasons were updated. For SARIMA forecasts, the model was fitted by the time series of dengue cases and used to forecast the same periods of dengue seasons recursively. The seasonal ARIMA model with *S* observations per period and *d* differencing passes, denoted by SARIMA(*p*,*d*,*q*)(*P*,*D*,*Q*)_*S*_, consist of two parts, an autoregressive part of order *p* and a moving average part of order *q*. Here, *P*, *D* and *Q* follow the same definition of *p*, *d*, *q* but are applicable to the seasonal component of the time series. In particular, we identified that the SARIMA(2,0,2)(1,1,0)_53_ model had the highest best-of-fit for the dengue incidence, which was automatically selected by the *auto*.*arima* function within the forecast R package. Therefore, the setting of the SARIMA model was used in our study.

**Fig 7:**
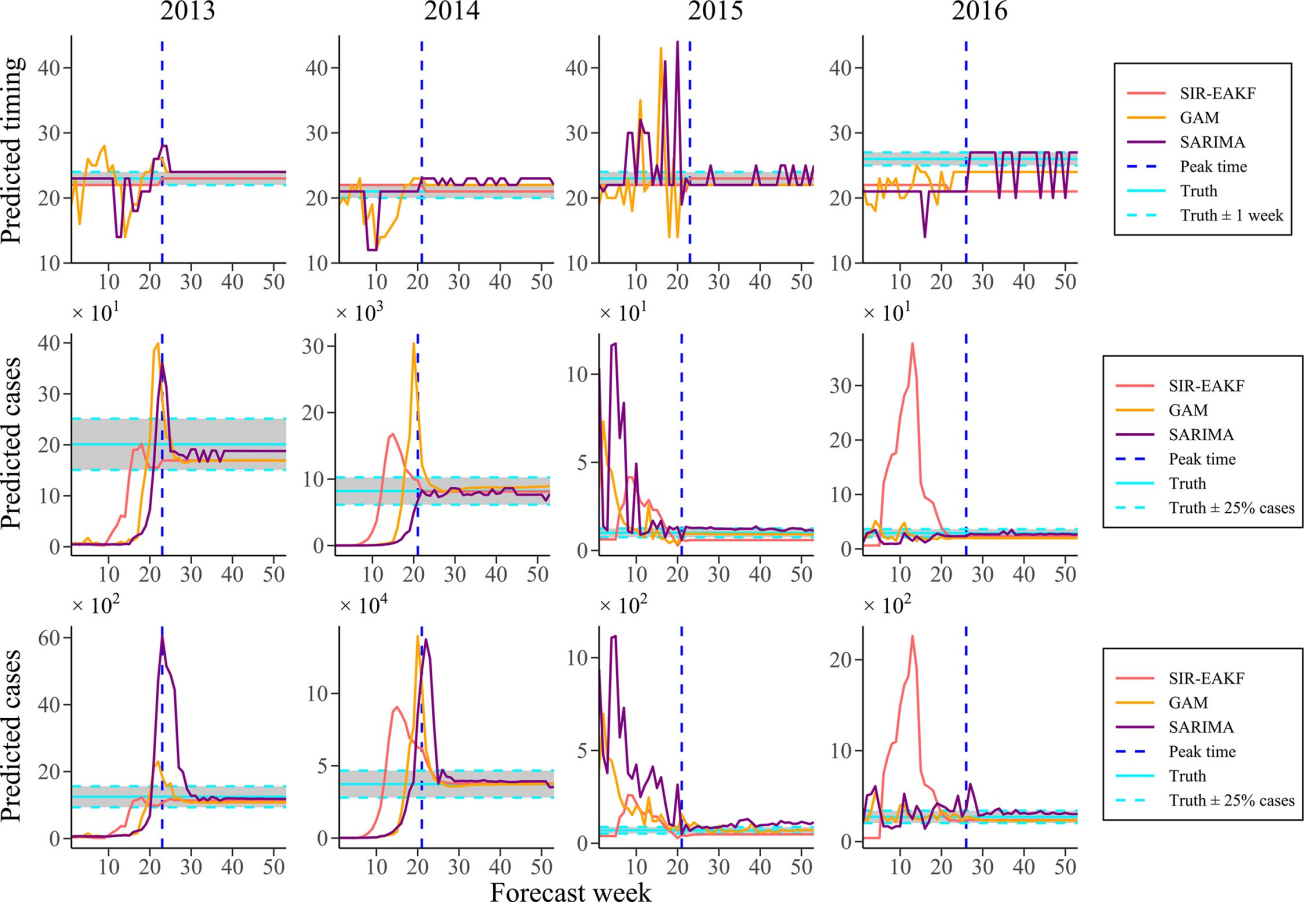
Comparison of the combined SIR-EAKF with the benchmark models in terms of several prediction targets (peak time, peak intensity and total incidence) from the seasons of 2013–2014 to those of 2016–2017. The peak time, peak intensity and total incidence are shown in sequence from top to bottom. The true index (horizontal light blue solid line) and its accuracy interval (horizontal light blue dotted line; ± 1 week / ± 25% cases of the observed) and the observed peak (vertical royal blue dotted line) were also shown. Note that combined SIR-EAKF forecasts (red), GAM forecasts (golden) and SARIMA forecasts (purple) to the left of vertical line were made prior to the peak and forecasts to the right were made after the true peak had passed.

Fig 7 shows the performance of the GAM, SARIMA and the combined SIR-EAKF framework in terms of the three targets during the 2013–2014 to the 2016–2017 dengue seasons. Overall, the combined SIR-EAKF framework outperformed the GAM and SARIMA, and the former can accurately predict the peak time of dengue fever spread more time in advance. In addition, regarding to peak intensity and total incidence, the forecast of SIR-EAKF was more accurate than the GAM and SARIMA. For both of GAM and SARIMA, they are slightly less capable of making accurate predictions ahead the peak of dengue season than the SIR-EAKF framework, meaning that they have insufficient early warning of dengue epidemics.

## Materials and methods

### Construction of dynamic model of dengue fever transmission

In our study, the SIR model modulated by local ambient temperature conditions was adopted to simulate dengue outbreak dynamics. The transmission of dengue fever between mosquitoes and humans was described in the SIR model [24,33]. Under the assumption of a perfectly mixed population, the compartmental model describing the dynamics of dengue transmission can be constructed by the following equations:

$$\frac{dS_{M}}{dt}=-\frac{\tau (t)\beta_{0}S_{M}I_{H}}{N_{H}}-\alpha S_{M}+\mu_{b}(t)(S_{M}+(1-U)I_{M})-\mu_{d}S_{M}$$ (6)

$$\frac{dI_{M}}{dt}=\frac{\tau (t)\beta_{0}S_{M}I_{H}}{N_{H}}+\alpha S_{M}+\mu_{b}(t)UI_{M}-\mu_{d}I_{M}$$ (7)

$$\frac{dNewI_{M}}{dt}=\frac{\tau (t)\beta_{0}S_{M}I_{H}}{N_{H}}+\alpha S_{M}+\mu_{b}(t)UI_{M}$$ (8)

$$\frac{dS_{H}}{dt}=-\frac{\tau (t)\beta_{0}S_{H}I_{M}}{N_{H}}$$ (9)

$$\frac{dI_{H}}{dt}=\frac{\tau (t)\beta_{0}S_{H}I_{M}}{N_{H}}-\frac{I_{H}}{D}$$ (10)

$$\frac{dNewI_{H}}{dt}=\frac{\tau (t)\beta_{0}S_{H}I_{M}}{N_{H}}$$ (11)

$$\beta (t)=\tau (t)\beta_{0}$$ (12)

where *S*_*M*_ is the number of susceptible mosquitoes, *I*_*M*_ is the number of infected mosquitoes, *NewI*_*M*_ is the number of new infected mosquitoes, *S*_*H*_ is the number of susceptible humans, *I*_*H*_ is the number of infected humans, *NewI*_*H*_ is the number of new infected human, *N*_*M*_ is the mosquito population, *N*_*H*_ is the human population, *α* is the rate of dengue seeding into the local model domain, *U* is the dissemination rate and is constant over an outbreak, *μ*_*b*_(*t*) is the mosquito birth rate at time *t*, *μ*_*d*_ is the mosquito death rate and is constant over an outbreak, *τ*(*t*) is the population transmission rate at time *t*, *β*_0_ is the basic contact rate between humans and mosquitoes, *β*(*t*) is the contact rate between humans and mosquitoes at time *t*, *D* is the mean infectious period for human. Since the observation data of *NewI*_*H*_ rather than *I*_*H*_ were used and the data needed to be assimilated in the filtering algorithm, this required us to calculate the prior value of the parameter based on the given formula of *NewI*_*H*_. The difference between *NewI*_*H*_ and *I*_*H*_ is that the former only refers to the number of newly infected people over a period, while the latter refers to the number of infected people currently present, specifically representing the number of people who left the infection state due to death or recovery based on *NewI*_*H*_.

Throughout the study, we set a constant population of humans with no birth or death and time-variant population of mosquitoes with the seasonal natural birth [29]. And the population transmission rate, which can be further explained the seasonality of dengue outbreak, is translated from the ambient temperature using linear regression piecewise function [27]. Besides, we consider vertical transmission of mosquitoes using a constant dissemination rate, *U*, to develop the transmission path of dengue fever. In addition, the simulation is seeded with infected mosquitoes at a rate of 1 in 500,000 susceptible mosquitoes [16]. Furthermore, several studies [34,35] have shown that dengue has a high under-reporting rate, but there are few studies on China. So, for model scaling, we assume that the number of dengue cases reported in clinics represent 35% of total new infections each week by model free simulation. Following Eqs 6–12 mentioned above, the compartmental model is then integrated forward using classical Runge-Kutta method, which can provide a more accurate prediction for state variables in each forecast (see the S1 Text for more details).

### Observational data

The individual surveillance data of dengue fever cases in Guangzhou from 2011 to 2018 were obtained from Guangdong Provincial Centers for Disease Control and Prevention (CDC). All human dengue cases were diagnosed according to the diagnostic criteria for dengue fever (WS216-2008) enacted by Chinese Ministry of Health [36,37]. Among them, dengue virus subtypes were divided into four types, all of which were included in the statistics. Only the local cases were included in this study to avoid the uncertainty of import cases. Dengue cases were aggregated by week according to the date of illness onset with each week defined as Sunday to Saturday. Fig 1B–1H shows the weekly observations of dengue cases from Jun 2011 to Dec 2017 in Guangzhou.

Mosquito density surveillance data using the MOI in Guangzhou from 2011 to 2018 were obtained from Guangdong CDC. The MOI is defined as number of ovitrap with positive adult and egg of Aedes albopictus/number of effective ovitrap [38]. Besides, daily meteorological data on ambient temperature, maximum temperature and rain volume for the same period were publicly available on the China Meteorological Data Sharing System (http://data.cma.cn/).

### An SIR type of compartmental model coupled with the EAKF algorithm

Previous studies [16,20,39] have used the EAKF assimilation algorithm in conjunction with a variety of compartmental epidemiological models to assimilate the observation data and update the simulation data based on Bayes’ rule, which also shows a better performance than other filtering methods [15,40]. EAKF adjusts ensemble of model simulation state variables to true state. Using cross ensemble co-variability, unobserved state variables and parameters are also updated. For further details of EAKF algorithm, see Anderson’s study [23]. In this study, a 300-member ensemble simulation of the dengue SIR compartmental model was run in conjunction with the Guangzhou dengue cases data and the EAKF. There were 6 state variables and 3 parameters $X_{t}=(S_{M},I_{M},S_{H},I_{H},D,\beta ,N,NewI_{M},NewI_{H})$ and weekly observations of human dengue cases $y_{t}^{o}=(NewI_{H})$ included in the filtering framework to estimate the true state and parameter of system.

The model assumed that the mosquito population size was time-varying for the combined affection of the seasonal birth rate and constant death rate. The EAKF consisted of 300-member ensemble of SIR model replicates each of which is initialized from a randomly drawn suite of state variable conditions and parameter values. The ensemble SIR model of dengue dynamics was used to predict the next state variables and then updated with EAKF on observation state variables. In addition, the inter-variable relationships were assumed to be linear. Consequently, a cross ensemble co-variability method was used to adjust the unobserved state variables and parameters $\left(S_{M},I_{M},S_{H},I_{H},D,\beta ,N,NewI_{M}\right)$ by multiplying the ensemble covariance with the observation adjustments in EAKF algorithm. The SIR-EAKF model was then integrated forward to the next observation using the updated (posterior) state variables and parameters and the data assimilation updating process was repeated. Over time, the observed dengue cases were used for recursive adjustment to optimize the model state variables and parameters, so that the integrated model simulation can better simulate the local outbreak dynamics.

If the ensemble spread tend to be too small due to long time iteration of filter adjustment, the EAKF system will diverge from the true trajectory, which is called ‘filter divergence’. In order to avoid filter divergence, a multiplicative inflation factor *λ* = 1.025 is used to expand the prior ensemble spread before assimilation. Besides, we introduced an observational error variance (OEV) in the algorithm to mimic observation error. Inspired by previous studies [13,19], we use a heuristic observational error variance (OEV) in running the EAKF, which consists of a baseline uncertainty and a proportional part determined by the new infection dengue cases on that week. Specifically, the OEV for week *t* is $OEV_{t}=(Obs_{t}{}^{2}+100)/25$.

### Effective population size and the combined SIR-EAKF

Due to the huge difference between peak intensity of each year, it is irrational to give a too large, fixed *N* for a dengue season with small peak intensity, and vice versa for a dengue season with large peak intensity. In practice, the expansion of the scale of transmission mostly caused by runaway epidemic, resulting in inflation of risk area and risk exposed population. With assumed the risk area is a fixed area with a constant radius centered on the disease source, the number of risk exposed population can be calculated by the population density of each area, which can be regard as the effective population size. Hence, from a perspective of biological significance, the effective population size actually represents the number of population affected by the epidemic in the risk region over time. Also the risk area as well as the effective population size is change over time. However, it was difficult to obtain the number of real-time risk exposed population. As a result, we consider the effective population size that varies with the observed time series of dengue cases to solve this problem.

In this study, in order to solve the problem of different scales of outbreaks in each epidemic season, we suggest using a simple estimation method for the *N*. The effective population size, *N*, was calculated from the historical data and used as a fixed parameter of SIR-EAKF. Then, the transmission model was rebuilt based on each update of effective population size by re-assimilating the observed data in this season. In particular, first of all, the average proportion of weekly new infected dengue cases (*obs*^*p*^) in the total number of infected dengue cases in that year was calculated according to the history data from 2011 to 2017 (except year of 2014 which have an abnormally high value; it can be seen from Fig 5B). After that, the value of *N* in current time was estimated through the average proportion sequence and the current observation by $N=\frac{c\times \sum_{i=1}^{t}{obs}_{i}}{scale\times \sum_{i=1}^{t}obs_{i}^{p}}$, where *scale* was the reported rate, *c* was a constant coefficient, which was used to multiply the minimum estimated population size to ensure a sufficient population size was provided. In this study, *c* was set as 12 and determined through simulation training. Finally, we calculated $N_{t}^{*}$ with a local smoothing by $N_{t}^{*}=\frac{N_{t-2}}{6}+\frac{N_{t-1}}{3}+\frac{N_{t}}{2}$ to reduce the noise and used as the updating effective population size. By estimating and updating *N* (the fixed parameter of SIR-EAKF) at time *t*, the assimilation and update process was needed to rerun the time period of {1,2,…,*t*}. Moreover, in the combined model, this method was set to run from the fifth week to the average peak time of 22 weeks to reduce the amount of computation, and the lower limit of the population size affected was 300 and the upper limit was 1,500,000.

### Retrospective forecasts

Guangzhou is one of the most prevalent areas of dengue fever in Guangdong Province, accounting for 78% of cumulative dengue cases reported from 2011 to 2018 in Guangdong Province. Therefore, Guangzhou is a representative location for analyzing the epidemic of dengue fever of Guangdong Province. We used an ensemble compartmental-model initiated with a 300-member ensemble to retrospectively forecast dengue epidemic for outbreak seasons of 2011–2012 to 2017–2018 in Guangzhou. Since the outbreak of dengue started in June and ended in December of that year, in order to reduce the filter divergence caused by long time adjustment of EAKF, a dengue season was moved forward and was defined as the 20th of week of each year to the 19th week of next year in running SIR-EAKF system. Each ensemble member state variables was initialized with: $S_{M}(0)=N_{M}(0)-I_{M}(0)$, $I_{M}(0)=U(0,N_{M}/1000)$, $S_{H}(0)=N_{H}\times U(0.7,0.9)-I_{H}(0)$, $I_{H}(0)=U(0,1)$; and model parameters were randomly selected from uniform distributions: D=U(5,7), $\beta_{0}=U(0.045,0.055)$, $N=N_{H}=N_{M}(0)=N_{p}/U(0.6,0.8)$, and average of peak time was set as the 22th week of the dengue season. The simulation was seeded with infected mosquitoes, *α*, during integration over season at a rate of 1 in 500,000. In addition, each ensemble member was initialized with constant model parameter: *U* = 0.25 and *μ*_*d*_ = 1/15 over an outbreak.

After retrospective forecast for each dengue season, the accuracy of ensemble forecasts was assessed using the three targets including peak timing, peak magnitude and total number of dengue cases over a season [13,15]. For all three targets, we compared the ensemble mean trajectory with observation. An accuracy ensemble forecast should meet the following conditions: 1) it peaked within ±1 week of the observed peak of new infected dengue cases; 2) the maximum new infected dengue cases was within ±25% or ±1 of the observed peak intensity; 3) the total number of new infected dengue cases over the entire season was within ±25% or ±1 of the total number of reported cases, whichever was larger. Besides, all forecasts were grouped by the same prediction lead and the fraction of accurate forecasts was quantified. In particular, the prediction lead meant that how many weeks in the future or past the outbreak peak was predicted to occur or to have occurred.

## Discussion

The main findings of the study demonstrated the performance of the combined model-EAKF, whose optimization of model ensemble simulation was repeated with an iteratively updated effective population size during data assimilation. The model can produce robust and accurate projections including estimates of unobserved states variable and adjustment of model parameters. With provision of updating effective population size, the ability of the SIR-EAKF framework for tracking the various scales of epidemic can be enhanced. Using the proposed framework, the designed dengue surveillance system can better inform medical and public health planning and intervention. For example, the real-time operational forecasts generated from the surveillance system can alert the public when dengue transmission risk is elevated and determine if further mosquito prevention measures should be adopted.

Regarding the seasonality of dengue fever, our study highlights population fluctuation on mosquitoes and activity of dengue fever in explaining the seasonality of dengue pandemic [41,42], which enhances the accuracy of peak time prediction to 85.7% with 6 or more weeks prior to the observed peak. In addition, the forecast of peak intensity and total incidence both have a good performance, leading to accurate forecast of 71.4% and 57.1% one week in advance respectively. These forecasts provide ample lead-time for undertaking targeted interventions. As a supplementary analysis, we also compared the combined SIR-EAKF model with baseline models, GAM and the SIR-EAKF framework. As shown in Fig 7, although the GAM forecast model assimilated more information including rainfall and more historical data, the combined SIR-EAKF model showed better performance, especially for the peak time predictions. Besides, without adjustment of effective population size, the SIR-EAKF framework struggled with different scales of dengue outbreak. For the prediction of three targets, the forecast accuracy of GAM varied temporally excelling over specific windows of time but working less optimally during other periods, while the forecasts of the SIR-EAKF framework tend to be biased for certain challenging outbreaks. Overall, the combined SIR-EAKF forecast system did a better job in simulating the characteristics of dengue outbreaks than other forecast systems. Moreover, we did not make a comprehensive comparison of the proposed model with all available forecast methods which may be more suitable but are not included in this study.

However, the prediction accuracy of the model is different in the high and low transmission seasons of dengue fever. Despite its good performance in tracking the peak time for dengue epidemic and outbreak, the model still has some difficulties in predicting low-case seasons when the incidence climbs sharply from zero to the peak, which is often observed in the time periods with peak intensity below 10. This is partly due to the greater impact of observation error on the fluctuation of reported dengue cases with increased dengue activity, or the excessively strict evaluation criteria of current targets to low transmission season. Nevertheless, the dengue prediction system has a good prediction for the season with high level of transmission observed, such as the 2014–2015 super dengue seasons. The study conducted by Oidtman et al. [41] considered that the super dengue outbreak that happened in 2014 was jointly affected by multiple factors, which was difficult to forecast accurately. Besides, the super dengue outbreak will cause detrimental impacts on public health. Therefore, for early warning of a similar super outbreak, it is more important to accurately simulate the dynamics trajectory during high transmission seasons of dengue fever, which can help reduce thousands of dengue cases and bring more potential benefits.

In order to simulate the transmission dynamics of dengue fever in population, the SIR type of compartmental model which incorporates the possibility of vertical transmission and horizontal transmission of dengue virus by vector Aedes mosquitoes was constructed. However, some unrepresented factors that may affect dengue transmission dynamics, such as the influence of environmental temperature [43] and humidity [44] as well as rainfall [45] on mosquito activities, different serotypes of dengue virus [46], imported cases [41], have not been fully considered partly because the data for the whole study region are not currently available. At present, limited by the available observation data, it is inappropriate to complicate the compartmental model into a high-dimensional model structure using only one observation data stream, which may be difficult to optimize. Only when more observed data streams are available, more complex compartmental models can be constructed to improve prediction performance. Nevertheless, given currently useful information we obtained, the compartment model we chose for the present work was capable of generating a realistic dengue outbreak in free simulation, and successfully described the seasonal characteristic of dengue. In the future, as more years of data become available, we hope to further validate and refine estimates of the peak timing, peak magnitude, and the total number of human dengue cases.

In addition, spatial diffusion is vital for understating the evolution of real-world complex dynamical transmission of mosquito-borne diseases. As shown in S2 Video, dengue epidemic spread from affected areas to surrounding areas during an outbreak, suggesting that there is an urgent need to develop a methodology for tracking the spatial spread of dengue outbreaks. The spatial diffusion of infectious diseases is caused by the movement of random visitors and recurrent commuters [47–49]. Therefore, further study should integrate the information into the SIR-EAKF framework. We will introduce a spatial metapopulation model by connecting each city based on human mobility, and forecast the spatial diffusion of dengue within a spatial network model [21]. These efforts will facilitate the transformation of dengue forecasting from point to space and its integration into decision-making.

## Supporting information

S1 Text Supporting information including: Runge-Kutta method for SIR model, filtering method of EAKF, generation of synthetic truth and observation, application of synthetic observations to the model-inference system, forecast procedure, robustness of the combined SIR-EAKF system, and references. (DOC)

S1 Fig Inference of state variables and parameters in the combined SIR-EAKF model. Prior (green) and posterior (red) mean estimates of state variables *S*_*M*_, *S*_*H*_, *I*_*M*_, *I*_*H*_, *NewI*_*M*_, *NewI*_*H*_ and parameters *D*, *β* and *N* as inferred by combined SIR-EAKF model, were displayed. The grey area is the spread of the ensemble forecast between the 25th and 75th percentile. The truth of outbreak (blue) was generated by the free simulation to represent the average weekly observations for the 2011–2012 through the 2017–2018 seasons except the 2014–2015 seasons. The synthetic observation (represented by the cross symbols) was computed by adding disturbance from the truth of weekly new infected dengue cases.
(PDF)

S2 Fig Weekly forecasts of peak timing based on the combined SIR-EAKF for the 2011–2012 through 2017–2018 seasons. The true timing of peak intensity occurs in the season (horizontal light blue solid line) and its accuracy interval (horizontal light blue dotted line; ± 1 week of the observed) and the observed peak (vertical royal blue dotted line) were also shown. Note that the combined SIR-EAKF forecasts (red) to the left of vertical line were made prior to the peak and forecasts to the right were made after the true peak had passed.
(PDF)

S3 Fig Weekly forecasts of peak intensity based on the combined SIR-EAKF for the 2011–2012 through the 2017–2018 seasons. The true peak intensity of the season (horizontal light blue solid line) and its accuracy interval (horizontal light blue dotted line; ± 25% or ± 1 cases of the observed) and the observed peak (vertical royal blue dotted line) were also shown. Note that the combined SIR-EAKF forecasts (red) to the left of vertical line were made prior to the peak and forecasts to the right were made after the true peak had passed.
(PDF)

S4 Fig Weekly forecasts of total incidence based on the combined SIR-EAKF for the 2011–2012 through the 2017–2018 seasons. The true total incidence of the whole season (horizontal light blue solid line) and its accuracy interval (horizontal light blue dotted line; ± 25% cases of the observed) and the observed peak (vertical royal blue dotted line) were also shown. Note that the combined SIR-EAKF forecasts (red) to the left of vertical line were made prior to the peak and forecasts to the right were made after the true peak had passed.
(PDF)

S5 Fig Sensitivity tests of the combined SIR-EAKF assimilation for changes to the time interval between observations. **a** Time series of the distributions of mean ensemble mosquito susceptible error relative to the synthetic truth for observations made every 2, 4, 6, 8, 10, and 12 days. 300-member EAKF assimilation runs were performed to generate each subplot. The box and whisker form shows the distribution of ensemble posterior mean error following each observation assimilation, including error median (red segment), 25th and 75th percentiles (blue box), extremes (whiskers), and outliers (pink cross) following each observation assimilation. For clarity, the box and whisker distributions are shown for every other assimilation for the 2-d time-step interval (*Top Left*). **b** Same as **a** but for mean ensemble new infected dengue cases. **c** Same as **a** but for mean ensemble human susceptible. **d** Same as **a** but for mean ensemble new infected mosquitoes. **e** Same as **a** but for the parameter *D* (mean infectious period). **f** Same as **a** but for the parameter *β* (contact rate).
(PDF)

S6 Fig Sensitivity tests of the combined SIR-EAKF assimilation for different ensemble sizes. Ensemble sizes tested are 20, 50, 100, 200, 500, and 1,000 members. The distribution of mean ensemble estimator for 250 EAKF assimilation runs for each of these ensemble sizes is shown at week 22 for *S*_*M*_, *S*_*H*_, *I*_*M*_, *I*_*H*_, *D*, *β*, *N*, *NewI*_*M*_ *and NewI*_*H*_.
(PDF)

S7 Fig Sensitivity tests according to different seasonal, time-varying OEV with weekly observed number of dengue cases for epidemic seasons during 2013–2014. The seasonal OEV is scaled by a multiple of 0.1, 0.2, 0.5, 1, 2, or 5. All runs use a 300-member ensemble and 7-d interval between observations. Each subplot shows the prior (red) and posterior (green) mean ensemble new infected dengue cases with different scaling of 250 EAKF assimilation along with observations (denoted by the blue cross symbols). Also, the mean spread of the ensemble forecast between the 10th and 90th percentile are shown in grey area.
(PDF)

S8 Fig Retrospective forecasts of other 9 cities in Guangdong province from the seasons of 2014–2015 to those of 2017–2018. All runs use a 300-member ensemble and 7-d interval between observations. Each subplot shows the prior (red) and posterior (green) mean ensemble new infected dengue cases along with observations (denoted by the blue cross symbols). Also, the mean spread of the ensemble forecast between the 10th and 90th percentile are shown in grey area.
(PDF)

S1 Video Animation of retrospective forecasts based on 300-member combined SIR-EAKF for 2013–2014 seasons. Ten weekly forecast of dengue cases for 2013–2014 season. The purple lines are the ensemble mean forecasts, the grey area is the spread of the ensemble forecast (light grey represents area between 10th and 90th percentile and the darker grey area represents the spread between the 25th and 75th percentile), blue lines are the ensemble mean posterior distribution, the green cross symbols are for data points assimilated into the model, and red star symbols denote future observations.
(MP4)

S2 Video The dynamics of dengue fever epidemics in Guangdong Province in 2014. The brown dots indicate dengue cases exist at the current time. This process assumes that the duration of dengue fever cases is 7 days. Besides, the current date and the cumulative number of dengue cases are recorded.
(MP4)
