# Supplementary material for: An ensemble forecast system for tracking dynamics of dengue outbreaks and its validation in China
Source: PLoS Comput Biol. 2022 Jun 27;18(6):e1010218. doi: 10.1371/journal.pcbi.1010218 (PMC9269975; doi:10.1371/journal.pcbi.1010218)
Supplement: S1 Text — (DOC) [file pcbi.1010218.s001.doc]

**Supporting Information**

S1 Text. Supporting information including: Runge-Kutta method for SIR model, filtering method of EAKF, generation of synthetic truth and observation, application of synthetic observations to the model-inference system, forecast procedure, **robustness of the combined SIR-EAKF system, and references.**

Runge-Kutta method for SIR model

In order to improve the accuracy of forward prediction of SIR model, the classical Runge-Kutta method was used in the iteration of state in SIR model. When estimating the slope $k$ of $x_{i-1}$ and $x_{i}$, it is obviously inaccurate to use only the slope $k_{1}$ of $x_{i-1}$, which will seriously increase the error. However, Runge-Kutta method is to estimate the slope values $k_{1},k_{2},...,k_{m}$ of several points on $[x_{i-1},x_{i}]$ and the weighted average of them is taken as the average slop of $y(x)$ on $[x_{i-1},x_{i}]$. Due to restrain the error by Runge-Kutta method, it can solve the iterative process of state variables more accurately when applied to the compartmental model. The classical Runge-Kutta algorithm used in this study is as follows:

$y_{i+1}=y_{i}+\frac{1}{6}h(k_{1}+2k_{2}+2k_{3}+k_{4})$ (1)

$k_{1}=f(x_{i},y_{i})$ (2)

$k_{2}=f(x_{i}+\frac{h}{2},y_{i}+\frac{1}{2}hk_{1})$ (3)

$k_{3}=f(x_{i}+\frac{h}{2},y_{i}+\frac{1}{2}hk_{2})$ (4)

$k_{4}=f(x_{i}+h,y_{i}+k_{3}h)$ (5)

where $k_{1}$ is the slope at the beginning of the time period; $k_{2}$ is the slope at the midpoint of the time period, and the slope $k_{1}$ is used to determine the value of *y* at the point $x_{i}+\frac{h}{2}$ by Euler method; $k_{3}$ is the slope at the midpoint of the time period, and the slope $k_{2}$ is used to determine the value of *y*; $k_{4}$ is the slope at the end of the time period, and its *y* value is determined by the slope $k_{3}$.

Filtering method of EAKF

The ensemble adjustment Kalman filter (EAKF) is a data assimilation technique which uses the surveillance data to recursively inform and optimize an ensemble of model simulations to improve the estimate of the true state and unobserved state variables and model parameters. The algorithm of EAKF is introduced below.

Kalman filter, in general, assume that the observations $y_{t}^{o}$ at time $t$ are allowed to be divided into several uncorrelated subsets $y_{t}^{o}=\{y_{t,k}^{o},k=1,...,K\}$. A joint state-observation vector for a given $t$ and $k$ can be written as $z_{t,k}=[X_{t},h_{t,k}(X,t)]$, where $h_{t,k}(X,t)$ is an observation operator and $X_{t}$ represents the state of model system at time $t$. The length of joint state vector is $n-m$, where $n$ is the size of $X_{t}$and $m$ is the size of the observational subset $y_{t,k}^{o}$. The estimated observation values can be calculated by equation$y_{t,k}=Hz_{t,k}$, where $H_{i,j}=1$ for $j=i+n$,$i=1,2,...,m$ and $H_{i,j}=0$ for all other elements. The conditional distribution after making use of the next subset of the joint state vector is

$p(z_{t,k}|Y_{t,k})=p(z_{t,k}|y_{t,k}^{o},Y_{t,k-1})$ (6)

where $Y_{t,k}$ represents a superset of $k$ observation subsets before time $t+1$. Returning to the approach of Jazwinski ^1^, Bayes’s rule gives

$p(z_{t,k}|Y_{t,k})=p(y_{t,k}^{o}|z_{t,k},Y_{t,k-1})p(z_{t,k}|Y_{t,k-1})/p(y_{t,k}^{o}|Y_{t,k-1})$ (7)

$p(y_{t,k}^{o}|z_{t,k},Y_{t,k-1})=p(y_{t,k}^{o}|z_{t,k})$ (8)

$p(z_{t,k}|Y_{t,k})=p(y_{t,k}^{o}|z_{t,k})p(z_{t,k}|Y_{t,k-1})/p(y_{t,k}^{o}|Y_{t,k-1})$ (9)

Equation (9) shows new sets of observations modify the prior joint state conditional probability distribution through the predictions based on previous observation sets.

Assuming that the observation error and prior distribution satisfy the Gaussian distribution, then the equation (9) can be represented by the convolution of two Gaussian distributions, and the distribution of result is still Gaussian distribution. So the covariance and mean of $p(z_{t,k}|Y_{t,k})$ can be written as

$\sum u=[(\sum p)^{-1}+H^{T}R^{-1}H]^{-1}$ (10)

$\bar{z}^{u}=\sum u[(\sum p)^{-1}\bar{z}^{p}+H^{T}R^{-1}y^{o}]$ (11)

where $R$ is the error covariance of observation $y^{o}=y_{t,k}^{o}$. A linear operator $A$ is introduced to get the updated ensemble.

$z_{i}^{u}=A^{T}(z_{i}^{p}-\bar{z}^{p})+\bar{z}^{u}$ (12)

EAKF adjusts ensemble of model simulation state variables to true state. Using cross ensemble co-variability, unobserved state variables and parameters are also updated. For further details of EAKF algorithm see Anderson ^2^.

Generation of synthetic truth and observation

We generated a synthetic, model-simulated dengue outbreak to validate EAKF and evaluation method for the effective population size optimization of dengue SIR model. This synthetic dengue outbreak was generated by free simulation of dengue SIR model and defined as the ‘truth’ ^3^. The simulation was initiated with$N_{M}(0)=N_{H}=12000/0.7$, $S_{H}(0)=0.8N_{H}$, $I_{H}(0)=0$, $S_{M}(0)=N_{M}-I_{M}(0)$, $I_{M}(0)=1.2$, $D=6$, $\beta_{0}=0.05$, $U=0.25$, $\mu_{d}=1/15$, $\alpha=1/500000$. The simulation generated by this parameter combination make a good representation of the mean reported weekly dengue cases for Guangzhou city from 2011 to 2017 except 2014. Synthetic observations of dengue cases were then generated by adding normally distributed random observational error (mean 0 and standard deviation equal to the observation × 0.025) to the truth ^3^. These synthetic error-laden observational records of reported dengue cases were then used for assimilation in the combined SIR-EAKF system. Besides, to improve the model adaptability for the tremendous difference between various types of dengue season, in this study, the Monte Carlo method is used to generate the seasonal error of $\tau(t)$ and $\mu_{b}(t)$. Each ensemble member’s seasonal variables of the mosquito birth rate ^4^ and population transmission ^5^ rate were generated by adding normally distributed random seasonal error (mean 0 and standard deviation equal to the standard error of the actual recorded weekly mosquito birth rate and population transmission rate from 2011 to 2018) to $\tau(t)$ and $\mu_{b}(t)$.

Application of synthetic observations to the model-inference system

Using the synthetic observations of weekly dengue cases and its defined OEV, the combined SIR-EAKF framework could show a performance on the estimation of the unobserved state variables and model parameters. In the combined SIR-EAKF system, a 300-member ensemble of model simulations was used in these optimization tests. The state variables of each ensemble member was initialized as: $S_{M}(0)=N_{M}(0)-I_{M}(0)$, $I_{M}(0)=U(0,N_{M}/1000)$, $S_{H}(0)=N_{H}\times U(0.7,0.9)-I_{H}(0)$, $I_{H}(0)=U(0,1)$; and model parameters were randomly selected from uniform distributions: $D=U(5,7)$, $\beta_{0}=U(0.045,0.055)$, $N=N_{H}=N_{M}(0)=N_{p}/U(0.6,0.8)$, and average of peak time was set as the 22th week of the dengue season ^6^. The simulation was seeded with infected mosquitoes, $\alpha$, during integration over season at a rate of 1 in 500,000. In addition, each ensemble member with the constant model parameter: $U=0.25$ and $\mu_{d}=1/15$ over an outbreak. Furthermore, several studies ^7, 8^ have shown that dengue has a high under-reporting rate, but there are few studies in China. So, for model scaling, we assumed that the number of dengue cases reported in clinics represent 35% of total new infections each week by model free simulation. For each outbreak, model optimization began from the 20th week of current year to the 19th week of next year. The dengue SIR model was run by day and the assimilation of synthetic observations of dengue cases was run by week using EAKF.

Overall, the ensemble posterior mean state variable and parameter estimates were well constrained (Supplementary Fig. 1). Affected by the time-varying effective population size, the estimation of susceptible mosquitoes $S_{M}$ and susceptible human $S_{H}$ is lower than the truth in the initial stage of the dengue season for a low epidemic-affected population size $N$. As time progresses and more information is observed about the outbreak, the unobserved state variables including $N$, $S_{M}$ and $S_{H}$ were well captured. Besides, the observed state variable, new infected human dengue cases $NewI_{H}$, and the unobserved state variables, new infected mosquitoes dengue cases $NewI_{M}$, infected human dengue cases $I_{H}$, infected mosquitoes dengue cases $I_{M}$, were also well captured over the dengue season. In addition, the combined model also estimated the epidemiological parameters which were helpful to describe the epidemic characteristics of infectious diseases. Specifically, the parameter $D$ (mean infectious period for human) fluctuates around the truth owning to the change of susceptible population. Due to the lack of initial susceptible population, model parameters $D$ were adjusted higher than the truth in response to the correction of observed variable. When an appropriate epidemic-affected population size was given in a re-run SIR-EAKF, the susceptible population was captured and the parameters $D$ were slightly adjusted closer to the truth. It was the same reason why the parameter $\beta_{0}$ (basic contact rate between humans and mosquitoes) fluctuates around the truth.

Forecast procedure

Weekly ensemble forecasts of future dengue cases are generated after assimilating the latest observation and updating the dengue SIR model. At the start of a particular dengue season, given a suitable set of initial conditions can help compartment model grasp the trend of outbreak and reduce the dependence on EAKF. In practice, the observations of weekly dengue cases are assimilated iteratively from the start of the simulation up to the point of forecast to further optimize the ensemble. By adjusting the ensemble of simulations repeatedly, the state variables and model parameter are better aligned to the observed outbreak so far this season, so as to make a more accuracy forecast of dengue cases. Using the latest posterior estimates of the state variable and model parameters, the compartmental model is integrated to generate those forecasts through time until the end of outbreak. In addition, during the 5th week to the 22nd week of dengue season, the SIR-EAKF system is rerun with an update effective population size.

**Robustness of the combined SIR-EAKF system**

In order to explore the robustness of the combined SIR-EAKF system, sensitivity analyses ^9^ were performed on observation interval, ensemble number and OEV (Supplementary Figures 5-7). For the sensitivity to time between observations, 300-member ensemble assimilations were performed for the same synthetic observations, but using observations made every 2, 4, 6, 8, 10 and 12 days. The start and the end of estimation method to effective population size were recalculated following the change of observation interval. Similarly, the interval of recorded MOI and meteorological temperature was correspondingly translated according to the time between observations by natural cubic spline. Supplementary Figure 5 shows that biases were relatively consistent across different setting of observation intervals. However, with the increase of observation interval, the character of the true outbreak becomes blurry. When the observation interval is shorter, the model can better capture the truth. Specifically, the EAKF can better align to the truth of $I_{M}$ and $I_{H}$ by high-frequency of observations. When the observation interval increases, the inter quartile range (IQR) of ensemble simulation increases correspondingly with the uncertainty of projection. Adjustment of model parameters $D$ and $\beta$ are both performed well in different observation interval situations. But for state variables $S_{M}$ and $S_{H}$, the longer the observation interval is, the smaller the IQR of ensemble simulation will be. It may be due to the decrease of observation assimilation times and the corresponding decrease of variance expansion times. Overall, the sensitivity of the combined SIR-EAKF framework to observation interval shows that the combined SIR-EAKF framework has stable prediction and correction ability under different observation interval settings.

In the same way, the same synthetic observation was used to conduct sensitivity analysis on the ensemble sizes of 20, 50, 100, 200, 500 and 1000. Each of these ensemble sizes combined SIR-EAKF model was run for 250 times with different initial conditions. In doing so, the distribution of mean ensemble estimator for state variables and model parameters in the 22nd week was displayed in Supplementary Figure 6. The certainty of prediction and the amount of computation increases with the number of ensemble members. Consequently, considering the balance between two of them, the number of ensemble was set as 300 in this retrospective analysis. The sensitivity to the prescribed OEV was also examined using observations of the 2013-2014 season, and are shown in Supplementary Figure 7. We respectively used values of 0.1, 0.2, 0.5, 1, 2, or 5 to multiply OEV to verify the dependence of the combined SIR-EAKF model on OEV. The results show that with the increase of OEV, the EAKF assimilation tends to follow the model simulation and deviates from the observation when the variance of ensemble is smaller than OEV. However, with the decrease of OEV, the variance of ensemble simulation is larger than the OEV during initial assimilation period. Thus, a greater weight on observation is given to make the ensemble simulations shrinking near the observation during the assimilation by EAKF, reducing the uncertainty of forecasts.

References

1. Jazwinski, A. H., 1970: Stochastic Processes and Filtering Theory. Academic Press, 376 pp.
2. Anderson, Jeffrey L . An Ensemble Adjustment Kalman Filter for Data Assimilation[J]. *Monthly Weather Review*, 2001, 129(12):2884-2903.
3. DeFelice, N. B., Little, E., Campbell, S. R., & Shaman, J. (2017). Ensemble forecast of human West Nile virus cases and mosquito infection rates. *Nature Communications*, 8, 14592.
4. Yang D, He Y, Ni W, Lai Q, Yang Y, Xie J, Zhu T, Zhou G, Zheng X. Semi-field life-table studies of Aedes albopictus (Diptera: Culicidae) in Guangzhou, China. *PLoS One*. 2020 Mar 18;15(3):e0229829.
5. Liu Z , Zhang Z , Lai Z , et al. Temperature Increase Enhances Aedes albopictus Competence to Transmit Dengue Virus[J]. *Front Microbiol*, 2017, 8:2337-.
6. Yamana T K, Kandula S , Shaman J . Superensemble forecasts of dengue outbreaks[J]. Journal of The Royal *Society Interface*, 2016, 13(123):20160410.
7. Carabali M, Lim JK, Palencia DC, Lozano-Parra A, Gelvez RM, Lee KS, Florez JP, Herrera VM, Kaufman JS, Rojas EM, Villar LA. Burden of dengue among febrile patients at the time of chikungunya introduction in Piedecuesta, Colombia. *Trop Med Int Health*. 2018 Nov;23(11):1231-1241.
8. Shankar MB, Rodríguez-Acosta RL, Sharp TM, Tomashek KM, Margolis HS, Meltzer MI. Estimating dengue under-reporting in Puerto Rico using a multiplier model. *PLoS Negl Trop Dis*. 2018 Aug 6;12(8):e0006650.
9. Shaman, J., &Karspeck, A. (2012). Forecasting seasonal outbreaks of influenza. *Proceedings of the National Academy of Sciences*, 109(50), 20425–20430.
