# Supplementary figures and images for: An ensemble forecast system for tracking dynamics of dengue outbreaks and its validation in China
Source: PLoS Comput Biol. 2022 Jun 27;18(6):e1010218. doi: 10.1371/journal.pcbi.1010218 (PMC9269975; doi:10.1371/journal.pcbi.1010218)

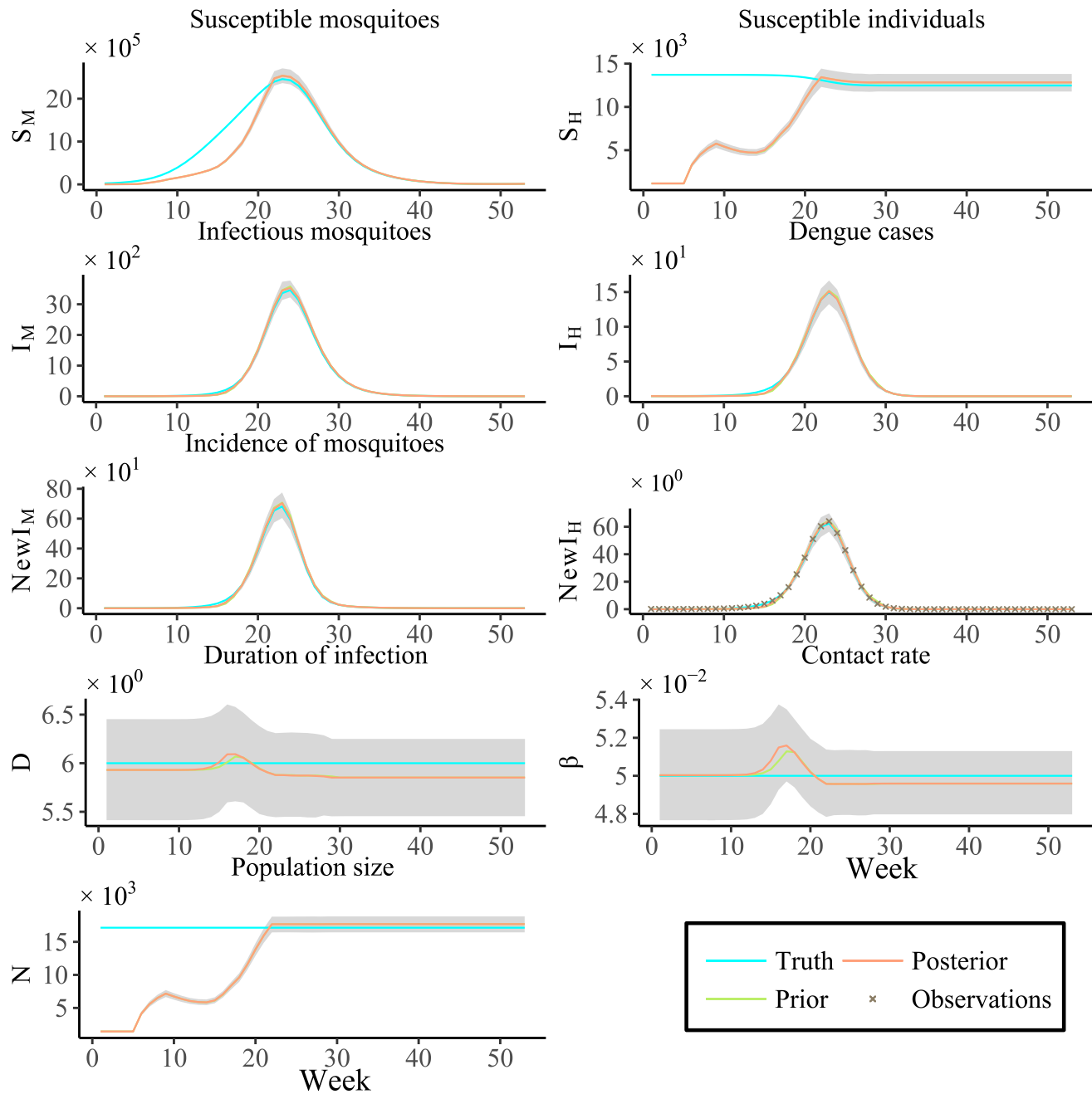

Supplement: S1 Fig — Prior (green) and posterior (red) mean estimates of state variables SM, SH, IM, IH, NewIM, NewIH and parameters D, β and N as inferred by combined SIR-EAKF model, were displayed. The grey area is the spread of the ensemble forecast between the 25th and 75th percentile. The truth of outbreak (blue) was generated by the free simulation to represent the average weekly observations for the 2011–2012 through the 2017–2018 seasons except the 2014–2015 seasons. The synthetic observation (represented by the cross symbols) was computed by adding disturbance from the truth of weekly new infected dengue cases. (PDF) [file pcbi.1010218.s002.pdf]

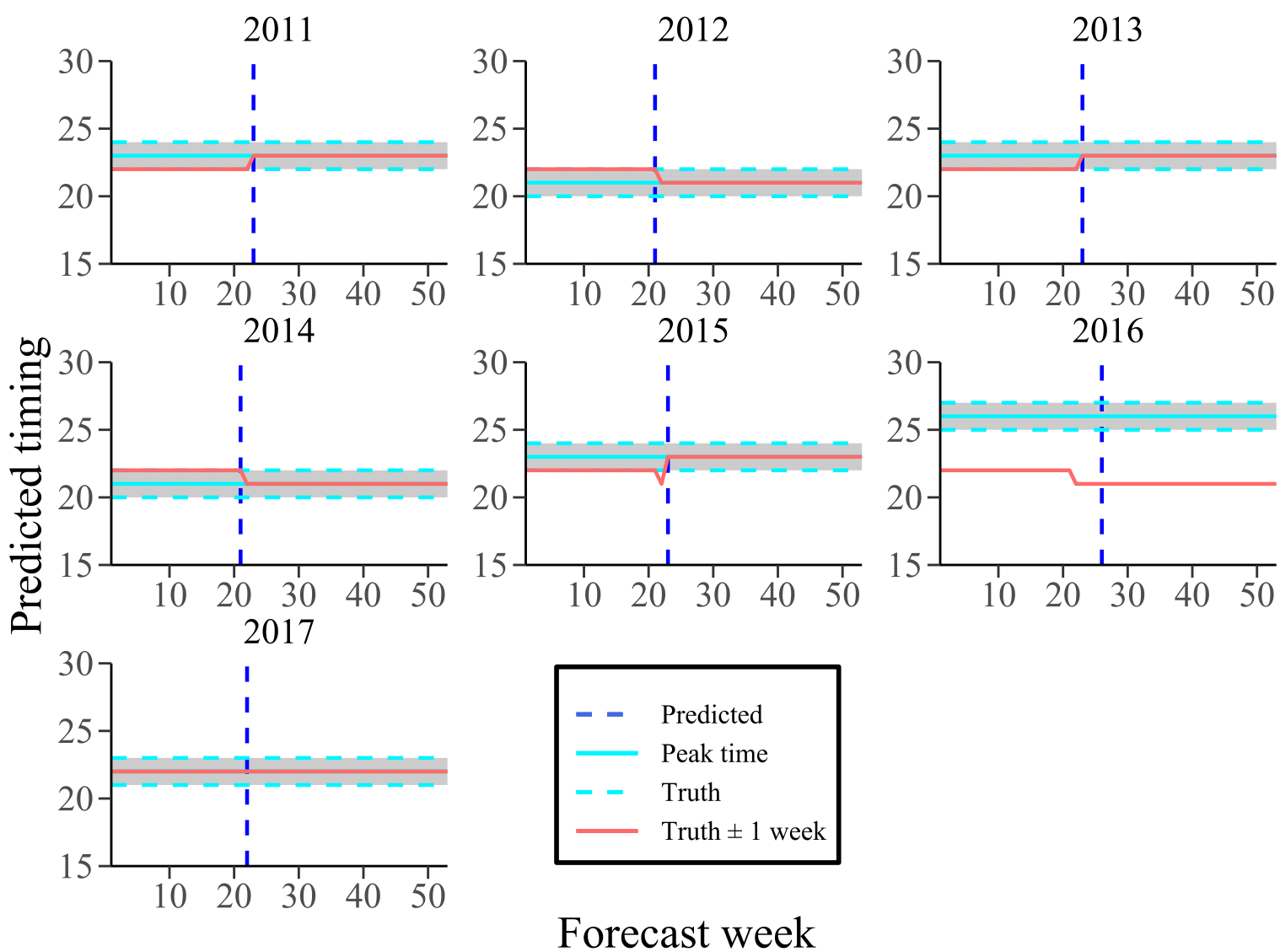

Supplement: S2 Fig — The true timing of peak intensity occurs in the season (horizontal light blue solid line) and its accuracy interval (horizontal light blue dotted line; ± 1 week of the observed) and the observed peak (vertical royal blue dotted line) were also shown. Note that the combined SIR-EAKF forecasts (red) to the left of vertical line were made prior to the peak and forecasts to the right were made after the true peak had passed. (PDF) [file pcbi.1010218.s003.pdf]

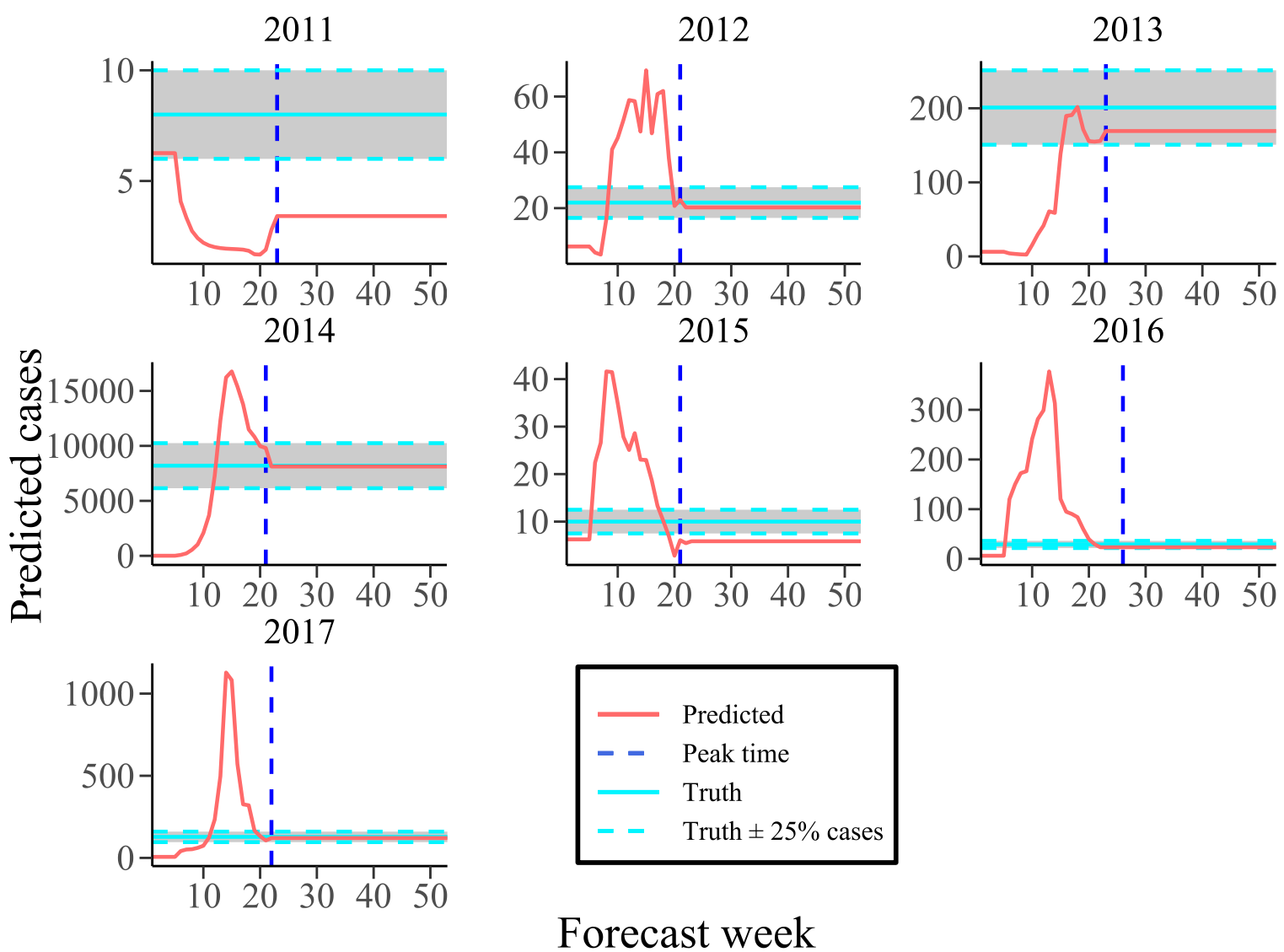

Supplement: S3 Fig — The true peak intensity of the season (horizontal light blue solid line) and its accuracy interval (horizontal light blue dotted line; ± 25% or ± 1 cases of the observed) and the observed peak (vertical royal blue dotted line) were also shown. Note that the combined SIR-EAKF forecasts (red) to the left of vertical line were made prior to the peak and forecasts to the right were made after the true peak had passed. (PDF) [file pcbi.1010218.s004.pdf]

Predicted cases

2011

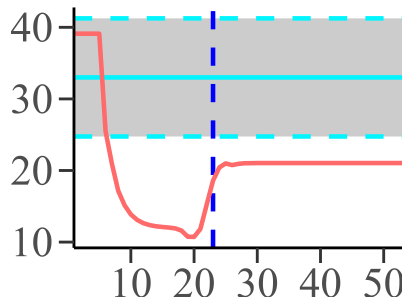

2012

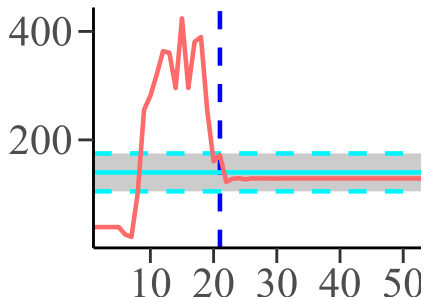

2013

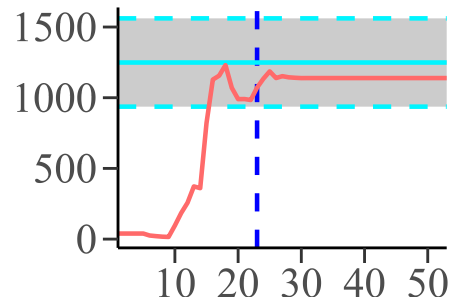

2014

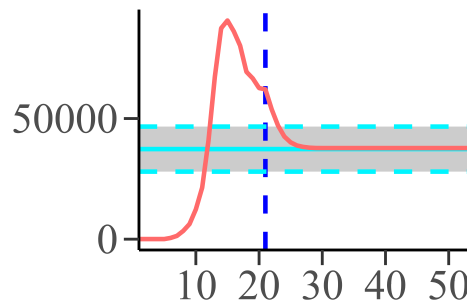

2015

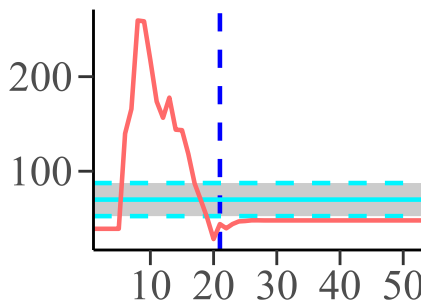

2016

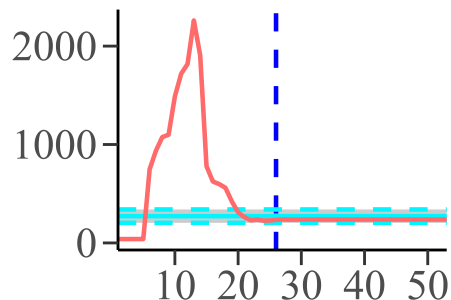

2017

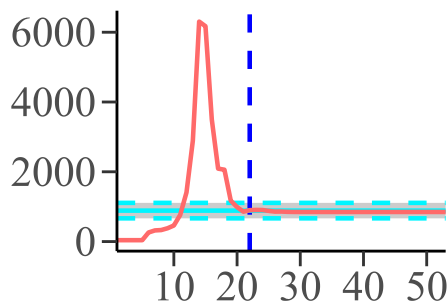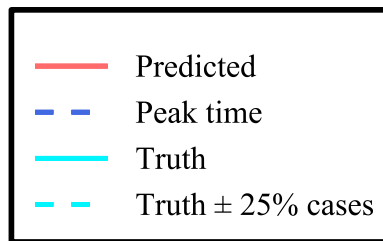

Forecast week

Supplement: S4 Fig — The true total incidence of the whole season (horizontal light blue solid line) and its accuracy interval (horizontal light blue dotted line; ± 25% cases of the observed) and the observed peak (vertical royal blue dotted line) were also shown. Note that the combined SIR-EAKF forecasts (red) to the left of vertical line were made prior to the peak and forecasts to the right were made after the true peak had passed. (PDF) [file pcbi.1010218.s005.pdf]

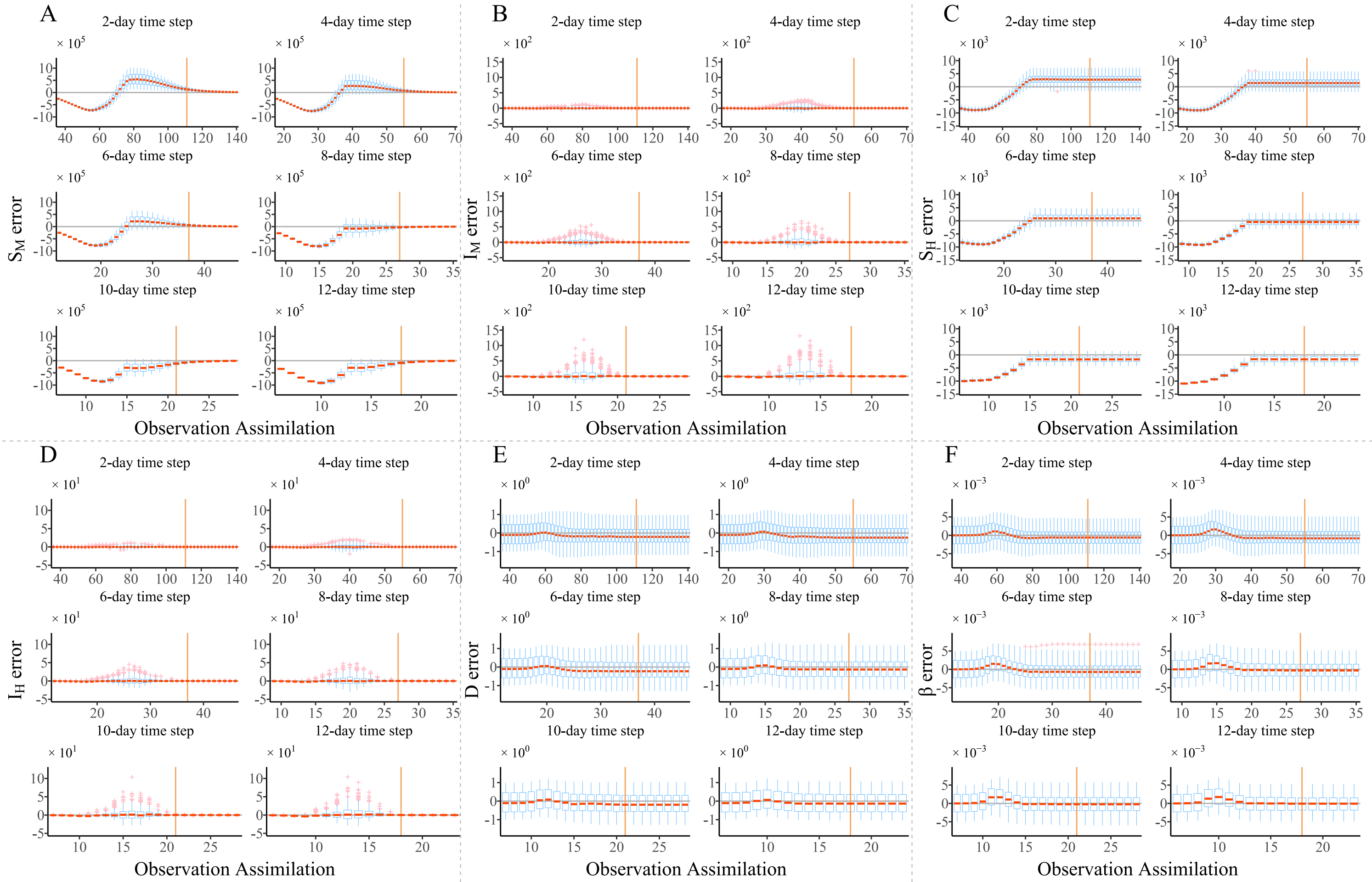

Supplement: S5 Fig — a Time series of the distributions of mean ensemble mosquito susceptible error relative to the synthetic truth for observations made every 2, 4, 6, 8, 10, and 12 days. 300-member EAKF assimilation runs were performed to generate each subplot. The box and whisker form shows the distribution of ensemble posterior mean error following each observation assimilation, including error median (red segment), 25th and 75th percentiles (blue box), extremes (whiskers), and outliers (pink cross) following each observation assimilation. For clarity, the box and whisker distributions are shown for every other assimilation for the 2-d time-step interval (Top Left). b Same as a but for mean ensemble new infected dengue cases. c Same as a but for mean ensemble human susceptible. d Same as a but for mean ensemble new infected mosquitoes. e Same as a but for the parameter D (mean infectious period). f Same as a but for the parameter β (contact rate). (PDF) [file pcbi.1010218.s006.pdf]

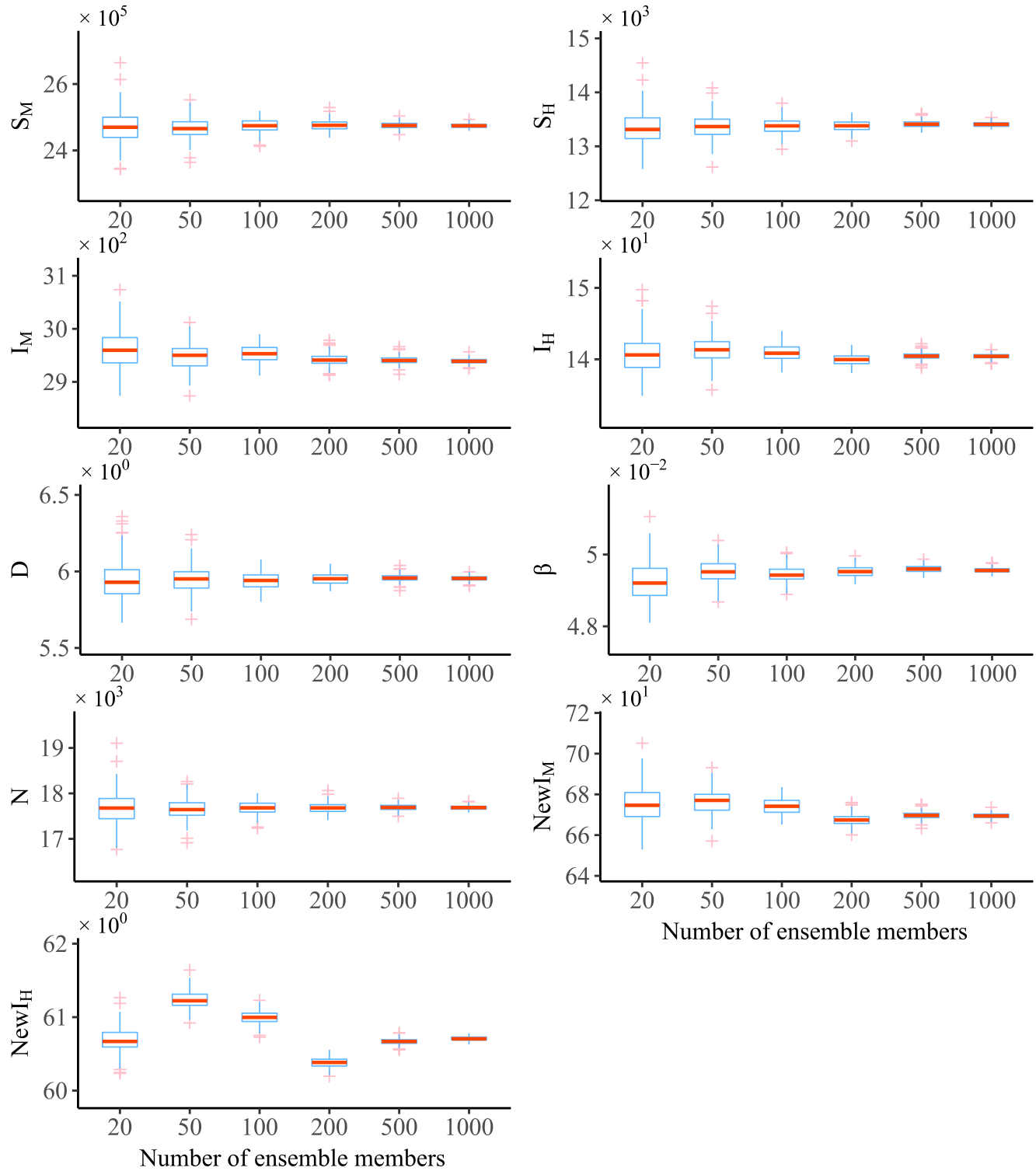

Supplement: S6 Fig — Ensemble sizes tested are 20, 50, 100, 200, 500, and 1,000 members. The distribution of mean ensemble estimator for 250 EAKF assimilation runs for each of these ensemble sizes is shown at week 22 for SM, SH, IM, IH, D, β, N, NewIM and NewIH. (PDF) [file pcbi.1010218.s007.pdf]

Dengue cases

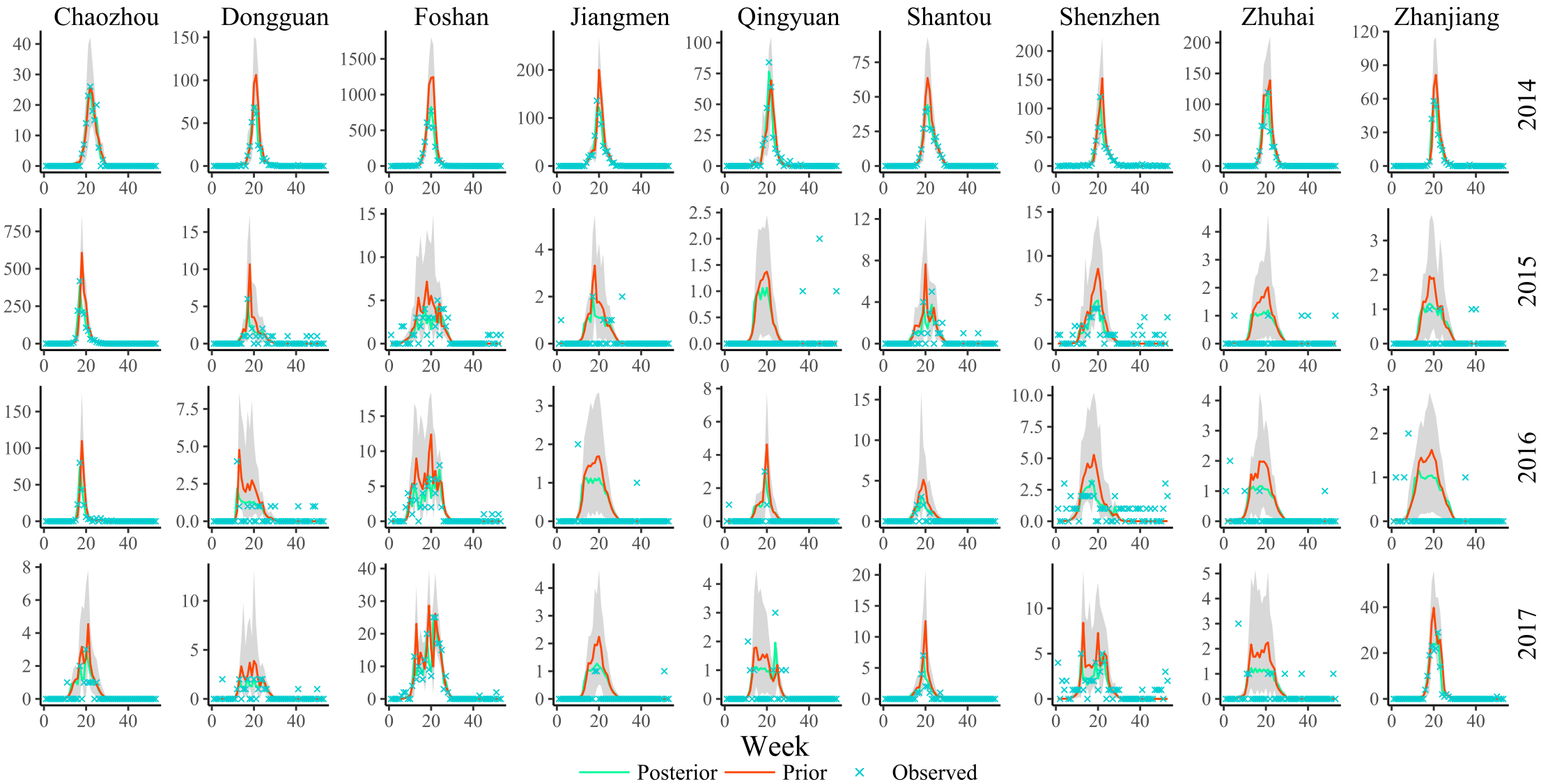

Supplement: S8 Fig — All runs use a 300-member ensemble and 7-d interval between observations. Each subplot shows the prior (red) and posterior (green) mean ensemble new infected dengue cases along with observations (denoted by the blue cross symbols). Also, the mean spread of the ensemble forecast between the 10th and 90th percentile are shown in grey area. (PDF) [file pcbi.1010218.s009.pdf]
