# Supplementary material for: An ensemble forecast system for tracking dynamics of dengue outbreaks and its validation in China
Source: PLoS Comput Biol. 2022 Jun 27;18(6):e1010218. doi: 10.1371/journal.pcbi.1010218 (PMC9269975; doi:10.1371/journal.pcbi.1010218)
Supplement: S7 Fig — The seasonal OEV is scaled by a multiple of 0.1, 0.2, 0.5, 1, 2, or 5. All runs use a 300-member ensemble and 7-d interval between observations. Each subplot shows the prior (red) and posterior (green) mean ensemble new infected dengue cases with different scaling of 250 EAKF assimilation along with observations (denoted by the blue cross symbols). Also, the mean spread of the ensemble forecast between the 10th and 90th percentile are shown in grey area. (PDF) [file pcbi.1010218.s008.pdf]

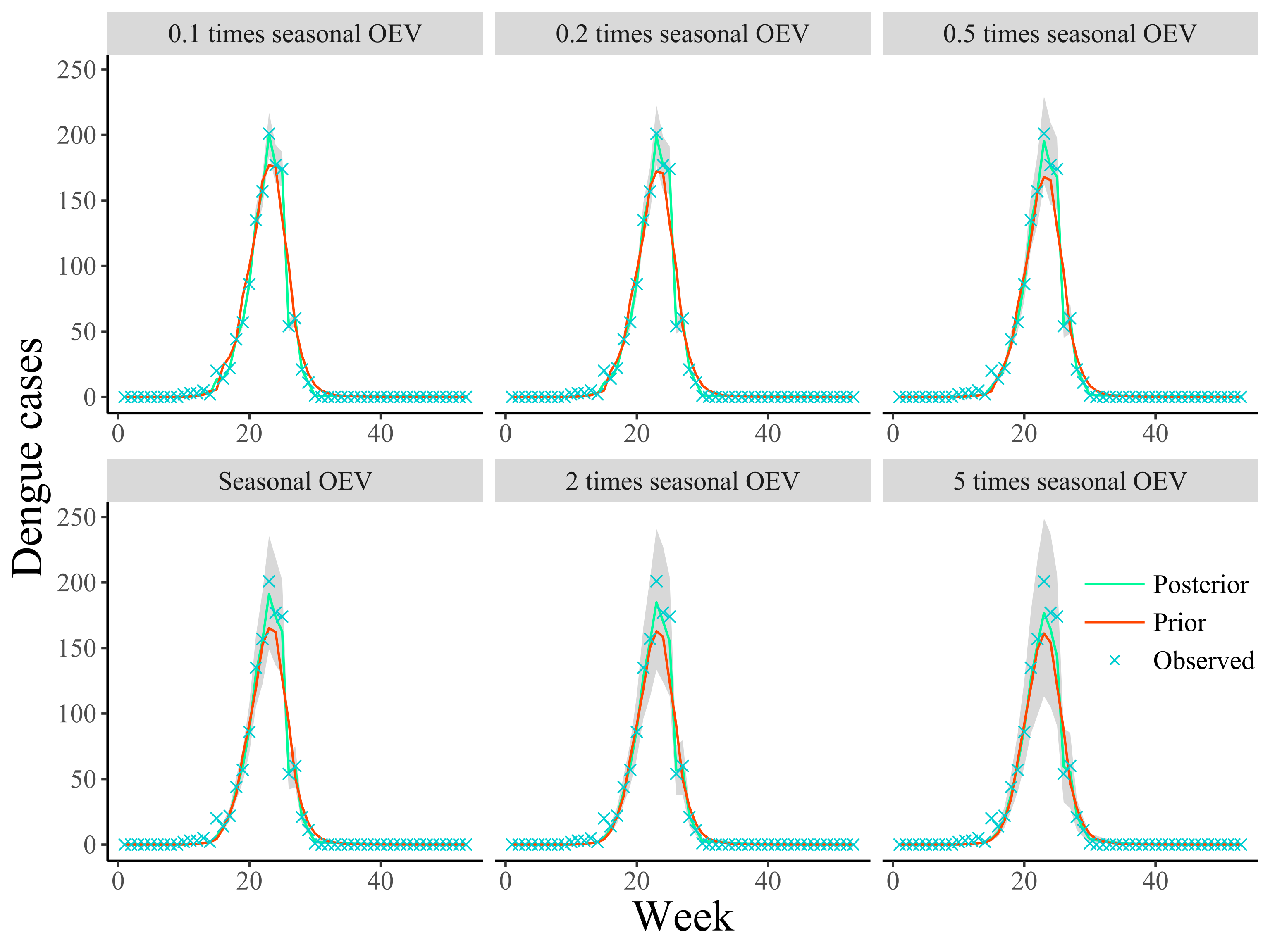
0.1 times seasonal OEV
0.2 times seasonal OEV
0.5 times seasonal OEV
Seasonal OEV
2 times seasonal OEV
5 times seasonal OEV
Dengue cases
Week
0
50
100
150
200
250
0
20
40
Posterior
Prior
Observed
